# De Novo Missense Mutations in *TNNC1* and *TNNI3* Causing Severe Infantile Cardiomyopathy Affect Myofilament Structure and Function and Are Modulated by Troponin Targeting Agents

**DOI:** 10.3390/ijms22179625

**Published:** 2021-09-06

**Authors:** Roua Hassoun, Heidi Budde, Hans Georg Mannherz, Mária Lódi, Setsuko Fujita-Becker, Kai Thorsten Laser, Anna Gärtner, Karin Klingel, Desirée Möhner, Robert Stehle, Innas Sultana, Thomas Schaaf, Mario Majchrzak, Verena Krause, Christian Herrmann, Marc M. Nowaczyk, Andreas Mügge, Gabriele Pfitzer, Rasmus R. Schröder, Nazha Hamdani, Hendrik Milting, Kornelia Jaquet, Diana Cimiotti

**Affiliations:** 1 Institut für Forschung und Lehre (IFL), Molecular and Experimental Cardiology, Ruhr University Bochum, 44801 Bochum, Germany; Roua.Hassoun@rub.de (R.H.); heidi.budde@rub.de (H.B.); Hans.Mannherz@ruhr-uni-bochum.de (H.G.M.); Innas.Sultana@ruhr-uni-bochum.de (I.S.); tschaaf98@web.de (T.S.); mario.majchrzak@rub.de (M.M.); Verena.Krause@rub.de (V.K.); andreas.muegge@ruhr-uni-bochum.de (A.M.); nazha.hamdani@rub.de (N.H.); 2 Department of Cardiology, St. Josef-Hospital and Bergmannsheil, University Clinic of the Ruhr University Bochum, 44801 Bochum, Germany; 3 Department of Anatomy and Molecular Embryology, Ruhr University Bochum, 44801 Bochum, Germany; 4 Department of Neuroanatomy and Molecular Brain Research, Medical Faculty, Ruhr University Bochum, 44801 Bochum, Germany; Maria.Lodi@ruhr-uni-bochum.de; 5 Cryoelectron Microscopy, Bioquant, Medical Faculty, University of Heidelberg, 69120 Heidelberg, Germany; Setsuko.Fujita-Becker@bioquant.uni-heidelberg.de (S.F.-B.); rasmus.schroeder@bioquant.uni-heidelberg.de (R.R.S.); 6 Centre for Congenital Heart Disease/Pediatric Cardiology, Heart and Diabetes Centre NRW, University Clinic of the Ruhr University Bochum, 32545 Bad Oeynhausen, Germany; tlaser@hdz-nrw.de; 7 Heart and Diabetes Centre NRW, Erich and Hanna Klessmann Institute, University Hospital of the Ruhr University Bochum, 32545 Bad Oeynhausen, Germany; agaertner@hdz-nrw.de (A.G.); hmilting@hdz-nrw.de (H.M.); 8 Institute for Pathology and Neuropathology, University Hospital Tuebingen, 72076 Tuebingen, Germany; Karin.Klingel@med.uni-tuebingen.de; 9 Institute of Vegetative Physiology, University of Cologne, 50931 Cologne, Germany; desiree.moehner@uk-koeln.de (D.M.); akq21@uni-koeln.de (R.S.); Gabriele.Pfitzer@uni-koeln.de (G.P.); 10 Department of Physical Chemistry I, Ruhr University Bochum, 44801 Bochum, Germany; chr.herrmann@rub.de; 11 Plant Biochemistry, Faculty of Biology and Biotechnology, Ruhr University Bochum, 44801 Bochum, Germany; marc.m.nowaczyk@rub.de; 12 Department of Clinical Pharmacology, Ruhr University Bochum, 44801 Bochum, Germany

**Keywords:** cardiomyopathy, pediatric, troponin mutation, skinned cardiomyocytes, skinned fibers, thin filaments, contractile function, levosimendan, EGCg

## Abstract

Rare pediatric non-compaction and restrictive cardiomyopathy are usually associated with a rapid and severe disease progression. While the non-compaction phenotype is characterized by structural defects and is correlated with systolic dysfunction, the restrictive phenotype exhibits diastolic dysfunction. The molecular mechanisms are poorly understood. Target genes encode among others, the cardiac troponin subunits forming the main regulatory protein complex of the thin filament for muscle contraction. Here, we compare the molecular effects of two infantile de novo point mutations in *TNNC1* (p.cTnC-G34S) and *TNNI3* (p.cTnI-D127Y) leading to severe non-compaction and restrictive phenotypes, respectively. We used skinned cardiomyocytes, skinned fibers, and reconstituted thin filaments to measure the impact of the mutations on contractile function. We investigated the interaction of these troponin variants with actin and their inter-subunit interactions, as well as the structural integrity of reconstituted thin filaments. Both mutations exhibited similar functional and structural impairments, though the patients developed different phenotypes. Furthermore, the protein quality control system was affected, as shown for TnC-G34S using patient’s myocardial tissue samples. The two troponin targeting agents levosimendan and green tea extract (-)-epigallocatechin-3-gallate (EGCg) stabilized the structural integrity of reconstituted thin filaments and ameliorated contractile function in vitro in some, but not all, aspects to a similar degree for both mutations.

## 1. Introduction

Pediatric cardiomyopathies (CM) are very rare heterogeneous disorders of the cardiac muscle, but they are usually characterized by a rapid disease progression with a poor prognosis and high mortality [1,2]. The most common CMs in children <18 years are dilated (DCM) and hypertrophic CM (HCM), whereas non-compaction (NCM) and restrictive CM (RCM) are the most uncommon ones. The assignment to a specific CM phenotype is complicated due to the frequent occurrence of mixed phenotypes [3,4,5,6,7]. CMs, especially idiopathic CMs, often are caused by point mutations, which may target genes encoding sarcomeric proteins. In pediatric CMs, de novo mutations seem to be more frequent and are linked to an especially severe disease development. Specific pharmacological treatments are still missing, especially for familial RCM and NCM; hence, heart transplantation is needed for survival [2]. Furthermore, the molecular mechanisms underlying the pathogenesis of RCM and NCM are not yet fully understood due to insufficient genetic testing and too few mechanistic studies. NCM is a structural disease, strongly affecting myocardial morphology and is mostly correlated to systolic dysfunction. In contrast, RCM is considered as a functional disease characterized by diastolic dysfunction, impaired relaxation, and increased myocardial stiffness. Both CMs are connected to malignant arrhythmia [2]. Furthermore, nearly all CMs are characterized by a disturbed protein quality control system, leading to increased protein aggregation, apoptosis, and fibrosis [8,9,10]. Structural and functional characterization of some RCM cases indicated the existence of mutation-induced myofilament dysfunctions, such as alterations of Ca^2+^-sensitivity, acto-myosin ATPase activity, and force generation. All these processes are prominently regulated by the heterotrimeric cardiac troponin complex (cTn), whose genes are frequently targets for RCM and NCM mutations, though no studies on the molecular level are available in case of NCM [2,11,12].

**Figure 1:**
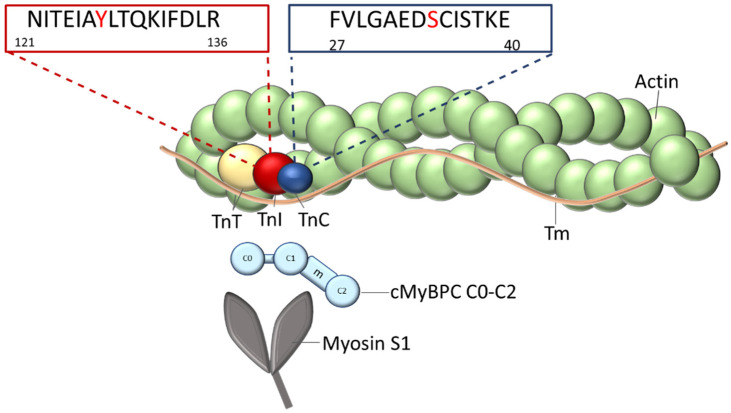
Organization of the thin filament: The thin filament is composed of the actin filament decorated with the elongated tropomyosin molecules (Tm) on both sides. Each Tm molecule spans seven actin subunits. At the end-to- end contacts of adjacent Tm-molecules, one troponin complex is bound, which is composed of TnT, TnI, and TnC subunits. The positions of the mutated amino acids within the sequences in the appropriate genes, *TNNI3* and *TNNC1*, are indicated. The N-terminal fragment of myosin binding protein C (cMyBPC C0-C2) and the myosin subfragment-1 (myosin-S1), which both interact with the thin filament, are shown.

cTn is associated with the thin filament and consists of three subunits (Figure 1). The tropomyosin binding subunit (cTnT) fixes the complex to the thin filament by binding to tropomyosin (Tm) with its N-terminal tail domain and is involved in the transmission of the Ca^2+^-signal to the thin filament [13,14,15,16]. The Ca^2+^-binding (cTnC) subunit contains four EF-hand Ca^2+^-binding motifs. The two C-terminal Ca^2+^-binding sites III and IV are Ca^2+^-unspecific high affinity sites; the N-terminal site I is non-functional but affects the Ca^2+^-binding affinity of the N-terminal site II, a low affinity regulatory Ca^2+^-specific binding site [17]. cTnC contains Ca^2+^-dependent and independent binding sites for the other troponin subunits. In the relaxed state of the cardiomyocytes, when cTnC is Ca^2+^-free, the inhibitory component (cTnI) binds to actin/tropomyosin with its regulatory C-terminal domain, thereby inhibiting the actin-myosin interaction and force production (also termed the blocked or B-state). Upon Ca^2+^-binding to cTnC, the switch domain of cTnI-C-terminus interacts with the N-terminal domain of cTnC. This is followed by the detachment of the mobile cTnI-C-terminus from actin/Tm. Consequently, Tm moves azimuthally on the actin filament first to the so-called closed (C-) state allowing a weak binding of few myosin heads. Subsequent binding of additional myosin heads pushes the Tm to the open-state (M-state), which allows strong binding of myosin heads and, thereby, force production.

There are specific genes encoding the cTnI and cTnT isoforms, whereas cTnC and slow skeletal muscle TnC are identical and are encoded by the same gene.

All genes encoding the cardiac troponin subunits are targets for cardiomyopathy inducing mutations, whereby *TNNI3*, encoding cTnI, is the main target for RCM mutations. In cTnI, most pathogenic modifications identified so far are located in its highly conserved C-terminus containing the inhibitory, switch, and mobile regions. Most pediatric RCM mutations are point mutations, leading to single amino acid replacements in this region of cTnI. In *TNNC1* encoding cTnC, only a few pathogenic modifications have been identified, mostly correlated to DCM or HCM [2], but at least 5 mutations have been described which induce non-compaction cardiomyopathy [12].

Here, we report a comparative mechanistic study of two de novo point mutations in *TNNC1* and *TNNI3* in infants younger than one year with end stage heart failure of NCM and RCM phenotype, respectively (Figure 1). In case of the *TNNC1* mutation, the new-born had an enlarged, fibrotic cardiac muscle and sponge-like ventricles. This child was transplanted shortly after diagnosis. In this patient’s cTnC, the glycine residue at position 34 in the non-functional Ca^2+^-binding site I was found to be substituted by serine (G34S). Therefore, it was conceivable that Ca^2+^ binding and/or cTnI binding of the mutated cTnC might be impaired. Thus, it seemed reasonable to analyze the functional consequences of this mutation in terms of cardiac myofilament Ca^2+^-sensitivity, as well as its influence on protein-protein interactions within the troponin complex and, subsequently, myofilament structure and stability. In addition, we assessed myocardial expression levels of, which are known to be key regulators of the protein quality control system, such as small heat shock proteins (sHsps), calpain, and cathepsin.

The point mutation in *TNNI3* leading to an exchange of an aspartate residue at position 127 by a tyrosine (D127Y) in cTnI was identified in an infant RCM patient who died shortly after diagnosis. This amino acid replacement is localized within the IT arm of cTnI (residues 42–136), followed by the inhibitory region (residues 137–148), the switch region (residues 149–164), and the mobile C-terminus (residues 165–210) [18]. Since this mutation affected protein–protein interactions with other thin filament proteins, we assumed that the inhibition of the acto-myosin ATPase could be impaired, as well as thin filament responsiveness to Ca^2+^. Our data show that, indeed, both mutants impair contractile function, the interplay of sarcomeric proteins, and the structural integrity of the thin filaments. In addition, histological and western blot analysis of patient’s myocardial tissue indicated that the cTnC-G34S mutation was accompanied by disturbances in the protein quality control system.

It is well established that heart transplantation is the only available treatment for severe pediatric cardiomyopathies. Without heart transplantation, the mean survival time after diagnosis is around 2–6 years. Thus, the development of specific drug therapies is of major importance. Agents that pharmacologically target sarcomeric proteins are used to correct aberrant myofilament Ca^2+^-sensitivity without affecting intracellular Ca^2+^-concentration. Therefore, we additionally investigated the effects of two troponin targeting agents (levosimendan and the green tea catechin (-)-epigallocatechin-3-gallate, EGCg) on the Ca^2+^-dependent activation of thin filaments containing the cTnC-G34S or cTnI-D127Y mutation. We also aimed to compare the responsiveness of the two mutants to both drugs.

Levosimendan, a Ca^2+^-sensitizer, has been approved for the treatment of heart failure. It enhances Ca^2+^-sensitivity in different ways; one of the suggested mechanisms is by binding to the N-terminal domain of cTnC and stabilizing its open conformation [19]. The green tea catechin (−)-epigallocatechin gallate (EGCg) has been shown to decrease Ca^2+^-sensitivity in myofilaments and to improve diastolic function in a HCM mouse model [20]. A previous study by Robertson et al. (2009) demonstrated that EGCg binds to the C-terminal domain of cTnC and interferes with the cTnC-cTnI interaction, resulting in weak anchoring of the C-terminal end of cTnC to the thin filament and, thus, decreasing the Ca^2+^-sensitivity [21]. We found that both drugs modulate contractile function in vitro, as well as the structural integrity of reconstituted thin filaments.

## 2. Results

### 2.1. Clinical Characterization

#### 2.1.1. Patient 1 (cTnC-G34S)

This patient was transferred to our center directly after birth because of cardiomegaly in the X-ray and clinical signs of cardiocirculatory distress presenting with cardiac failure due to impaired biventricular function under catecholamine support (Figure S1.1). Family history was negative (Figure S1.3). Cardiac imaging with echocardiography and catheterization revealed signs of severely dilated ventricles with impaired systolic, as well as diastolic, function. Apical left ventricular regions with hypertrabeculation suggested non-compaction cardiomyopathy in stage 4 according to New York Heart Association guidelines (NYHA4). Myocardial biopsy neither resulted in typical findings of myocarditis nor dilated cardiomyopathy. Histology was consistent with endocardial fibroelastosis and distinctive microangiopathy. The patient was listed for cardiac transplant. Medical catecholamine therapy had to be intensified, including 4 courses of levosimendan, and carnitine was also substituted. After 10 weeks, he was successfully transplanted with a good postoperative course and is still in a good condition, after 5 years now. The explanted heart was characterized by hypertrabeculation and fibrosis (Figure S1.1.E).

#### 2.1.2. Patient 2 (cTnI-D127Y)

This 8-month-old boy was diagnosed with signs of heart failure due to restrictive physiology of both ventricles. Hepatomegaly and signs of pulmonary hypertension in the tricuspid regurgitant jet were present. Echocardiographic findings and hemodynamic testing in the catheterization laboratory revealed predominantly impaired biventricular diastolic function and enlarged atria. Since the conservative treatment was not successful, ending up in clinical deterioration to NYHA4, a biventricular pulsatile ventricular assist device (VAD) had to be implanted. Due to aortic, as well as mitral, incompetence, additional complications arose during the further course. Finally, the child died 7 months later on the waiting list for transplant due to complications of the VAD therapy. The family history was negative (Figures S1.1 and S1.3).

#### 2.1.3. Sequencing

Next, generation sequencing and variant screening of 174 genes associated with inherited cardiac conditions revealed two de novo likely pathogenic point mutations in *TNNC1* and *TNNI3*, genes encoding cTnC and cTnI, respectively, in the infants <1 y with end stage heart failure of NCM and RCM phenotype, respectively. The nucleotide substitution G ˃ A was detected in *TNNC1*, leading to amino acid exchange Gly ˃ Ser at position 34 incTnC, while a G ˃ T substitution in *TNNI3* resulted in the exchange Asp ˃ Tyr at position 127 of cTnI (Figure S1.2). The additional variants detected were of unknown significance, and no proofs for pathological significance were available (Tables S1.1 and S1.2). No sarcomeric mutations were detected in the family members of both patients (Figure S1.3).

#### 2.1.4. Histology

##### Patient 1 (cTnC-G34S)

Masson trichrome staining of the myocardium revealed a mild diffuse interstitial fibrosis. Disturbances of the intracellular structure including perinuclear loss of myofibrils were obvious in the heart of in this patient. Interestingly, as demonstrated by immunohistochemistry, troponin C expression was significantly reduced in some cardiomyocytes leading to an irregular distribution of cTnC throughout the myocardium (Figure S1.4).

##### Patient 2 (cTnI-D127Y)

Histology revealed a significant interstitial fibrosis in Masson trichrome staining. Hematoxylin/eosin staining suggested structural disturbances between the cardiomyocytes. However, in contrast to the other patient, troponin I was more homogeneously distributed in the myocardium (Figure S1.4).

### 2.2. Impact of p.cTnC-G34S and p.cTnI-D127Y on Contractile Function

**Figure 2:**
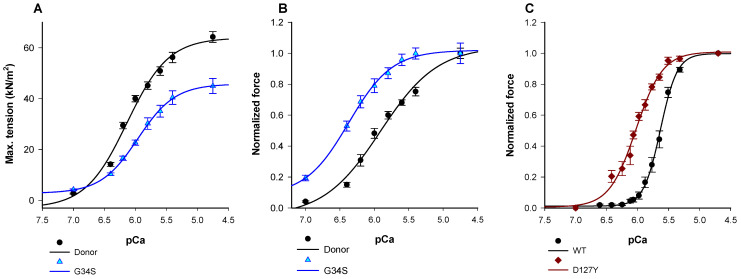
(**A**) Maximum tension of donors and cTnC G34S cardiomyocytes. (**B**) Ca^2+^- sensitivity of donors and TnC-G34S cardiomyocytes. (**C**) Force-pCa relation of guinea pig skinned fibers after exchanging endogenous troponin with human cardiac troponin containing either wild type cTnI or cTnI-D127Y. Data in A are shown as mean ± SEM, (*n* = 13/4 cardiomyocytes/heart for donor and *n* = 13 for G34S) vs. pCa (negative logarithm of the free Ca^2+^-concentration). Data in B are presented as normalized force ±SEM, (*n* = 13/4 cardiomyocytes/heart for donor and *n* = 13 for G34S) vs. pCa (negative logarithm of the free Ca^2+^-concentration). Data in C are presented as normalized force ±SEM, *n* = 6 for cTnI-WT fibres, and *n* = 4 for cTnI-D127Y fibres vs. pCa (negative logarithm of the free Ca^2+^-concentration).

We first aimed to investigate the impact of troponin mutations on the force generation and Ca^2+^-sensitivity. Therefore, we measured myofilament force generation in skinned cardiomyocytes isolated from frozen tissue of the explanted heart of the cTnC-G34S patient. The force-pCa (negative logarithm of the Ca^2+^ concentration) relation was shifted to the left by about ΔpCa_50_ = 0.4 units in cTnC-G34S cardiomyocytes (Figure 2, Table 1) in comparison to cardiomyocytes from a donor heart, indicating an increased myofilament Ca^2+^-sensitivity. Thus, pCa_50_ was significantly higher in cTnC-G34S samples (Table 1). In addition, the maximal active tension in cTnC-G34S cardiomyocytes was decreased significantly compared to controls.

The increased Ca^2+^-sensitivity of cTnC-G34S could be due to an altered Ca^2+^-affinity of cTnC-G34S. Therefore, the Ca^2+^ binding at the regulatory site II of cTnC-G34S was tested (Figure S3.1 and supplementary methods). For this purpose, we monitored the change in fluorescence intensity of 2-[4’-(iodoacetamido)anilino]naphthalene-6-sulfonic acid (IAANS) labeled troponin C subunits upon Ca^2+^-titration in thin filaments reconstituted with either cardiac troponin-G34S (cTn-G34S) or wild-type cardiac troponin (cTn-WT). Consistent with the results obtained from force recordings, the mutant filaments showed a significantly increased Ca^2+^-binding affinity compared to the respective wild type (Figure S3.2). The fluorescence-pCa curve was shifted to the left by a ΔpCa_50_ = +0.72. The determined pCa_50_ values were: Thin filament-G34S (TF G34S) = 7.49 ± 0.27, TF WT = 6.82 ± 0.14.

Most RCM mutations in cTnI investigated so far enhance the Ca^2+^-sensitivity of the force-Ca^2+^ relationship. Due to the unavailability of tissue from the cTnI-D127Y patient, we measured the Ca^2+^-sensitivity in guinea pig skinned fibers after exchanging about 60% of the endogenous cardiac troponin by cTnI-D127Y or cTnI-WT (Figure S3.2). We found that the pCa_50_ value was shifted to the left by about 0.5 pCa units (Table 1, Figure 2A). In contrast to cTnC-G34S, maximal force obtained with cTnI-D127Y was slightly, but not significantly, reduced compared to controls (Table 1, Figure S3.2).

In addition, we analyzed the Ca^2+^-sensitivity in vitro using reconstituted thin filaments. In a first approach, we investigated whether the formation of thin filaments was affected by the two mutants using co-sedimentation. No effect on the incorporation of cTnI or cTnC mutants into thin filaments was observed in comparison to wild type cTn subunits (Figure S3.3).

According to our findings on the pivotal role of cMyBPC in regulating myofilament kinetics [22], we performed all functional in vitro assays in the presence of the N-terminal cMyBPC C0-C2 fragment, which had been shown to interact directly with actin and troponin [22,23].

**Figure 3:**
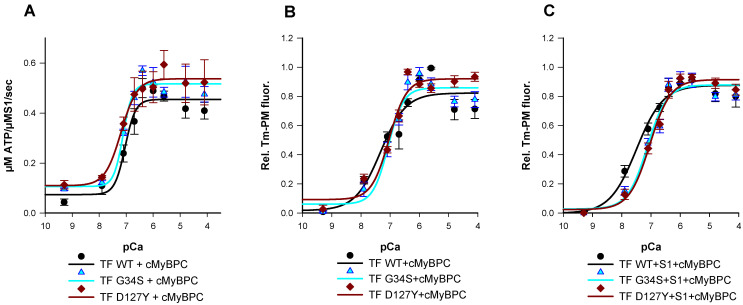
(**A**) Ca^2+^-dependent stimulation of the acto-myosin-S1-ATPase activity of thin filaments (TF) reconstituted with troponin complexes (Tn-WT, Tn-G34S, or Tn-D127Y), skeletal actin, tropomyosin, and cMyBPC C0-C2 as a function of pCa. Data points represent the rate of ATP hydrolysis by myosin-S1 as mean ± SEM, (*n* = 5–8). (**B**) Representative data for the Ca^2+^ dependent increase in fluorescence intensity of thin filaments reconstituted with troponin complexes (Tn-WT, Tn-G34S or Tn-D127Y), skeletal actin, pyrene-maleimide-labeled tropomyosin (Tm-PM), and cMyBPC C0-C2. (**C**) Representative data for the Ca^2+^ dependent increase in fluorescence intensity of thin filaments (TF) reconstituted with troponin complexes (Tn-WT, Tn-G34S, or Tn-D127Y), Tm-PM, cMyBPC, and myosin-S1. Data points are represented as mean of normalized fluorescence ± SEM vs. pCa and fitted to the Hill equation to obtain pCa_50_ and nHill values (*n* = 6–9).

The Ca^2+^-dependency of the acto-myosin-S1-ATPase activity was analyzed using an β-Nicotinamid-adenin-dinucleotid (NADH) enzyme-linked assay (Figure 3A). Thin filaments containing cTnC-G34S or TF containing cTnI-D127Y showed only marginal increase of their pCa_50_ or Hill slope compared to wild type TF (Table S2.1). Furthermore, the mutant filaments showed slightly but not significantly increased minimal activation rates compared to wild type filaments, indicating a potentially reduced inhibition of basal ATPase activity at low Ca^2+^ concentration (Table 2). However, compared to the respective wild type filaments, the mutant filaments showed a significant increase in the maximal ATPase activity, indicating a significant increase of the ATP-hydrolysis rate (TF-G34S Δy_max_ = +0.062 or 13.6%, TF-D127Y Δy_max_ = +0.082 or 18%) (Figure 3A, Table 2).

Next, we aimed to investigate whether the alterations in force production and acto-myosin-S1-ATPase kinetics are reflected by changes of thin filament activation. For this purpose, we monitored the Ca^2+^-dependent excimer fluorescence intensity of pyrene labeled tropomyosin (Tm-PM).

The transition from inactive to myosin-bound active state was measured after the incubation of Tm-PM, Tn, cMyBPC C0-C2, and myosin-S1 with F-actin in a 1:1:1:1:6 molar ratio. Pyrene–tropomyosin excimer fluorescence was Ca^2+^-dependent and increased upon Ca^2+^-saturation (Figure 3B). When measured in the absence of myosin-S1, the mutant filaments exhibited a slight but non-significant reduction in pCa_50_ values and no significant effects on thin filament co-operativity were obtained (Figure 3B, Table 3). However, in the presence of myosin-S1, a rightward shift in pCa_50_ of both TF-D127Y and TF-G34S towards lower values was detected compared to their respective wild type, indicating a lower Ca^2+^-sensitivity (TF-G34S ΔpCa_50_ = −0.37, TF-D127Y ΔpCa_50_ = −0.45) (Figure 3C).

Thus, the Ca^2+^-dependency of thin filament activation, cross-bridge cycling kinetics, and force production in skinned fibers or skinned cardiomyocytes were altered by both mutants, though the direction of the observed changes was dependent on the organization level of the model system and type of experiment used.

The observed contractile dysregulations might originate from altered protein-protein interactions. Therefore, we investigated the interactions of the troponin complex with actin and of troponin subunits among each other using microscale thermophoresis (MST).

### 2.3. Protein-Protein Interactions

**Figure 4:**
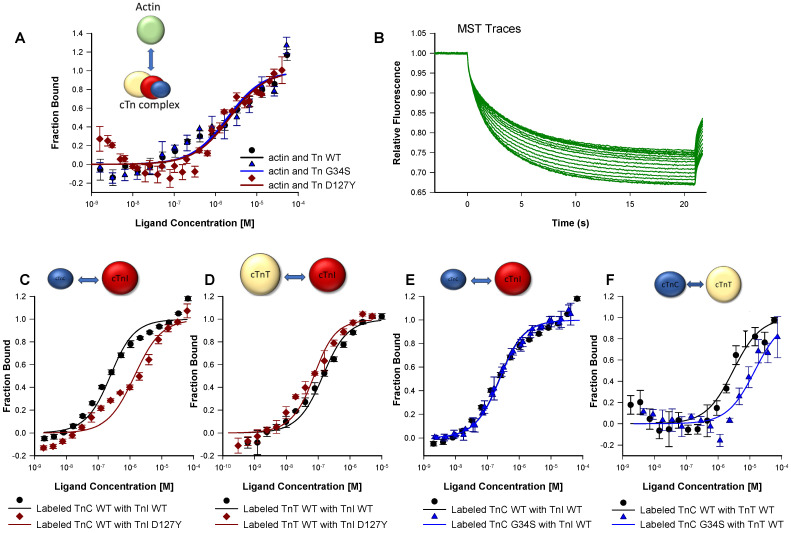
Protein₋protein interactions using MST. (**A**) Measurements of the interaction between fluorescently labeled actin and troponin complexes (Tn-WT, Tn-G34S, or Tn-D127Y). (**B**) Example of MST traces resulting from changes of the thermophoretic movement of the fluorescently labeled molecules upon binding to a ligand. (**C**) Measurements of the interaction of fluorescently labeled TnC-WT with either TnI-WT or TnI-D127Y. (**D**) Measurements of the interaction of fluorescently labeled TnT-WT with either TnI-WT or TnI-D127Y. (**E**) Measurements of the interaction of TnI-WT with either labeled TnC-WT or labeled TnC-G34S. (**F**) Measurements of the interaction of TnT-WT with either labeled TnC-WT or labeled TnC-G34S. In all measurements, the fraction bound was plotted vs. ligand concentration, and the resulting sigmoidal curves were fitted to the K_d_ model yielding the k_d_ values (*n* = 4–7).

The K_d_ values obtained with mutant troponin complexes and actin were comparable to those of the respective wild type (cTn-WT K_d_ = 2.01 ± 0.70 µM, cTn-G34S K_d_ = 1.93 ± 0.96 µM, cTn-D127Y K_d_ = 2.20 ± 0.68 µM), indicating that the troponin mutations did not change the binding affinity of the complexes towards actin (Figure 4A, Table S2.3). This finding agrees with the unchanged incorporation of the troponin variants into thin filaments as observed by co-sedimentation (Figure S5).

The binding experiments of the mutant troponin subunit cTnI-D127Y with cTnC yielded significantly reduced K_d_ values compared to cTnI-WT (cTnI-D127Y K_d_ =1.32 ± 0.41 nM, cTnI-WT K_d_ = 179.57 ± 47.22 nM). Hence, the affinity of cTnI-D127Y towards cTnC was significantly higher than that of wild type cTnI (Figure 4C, Table S2.2). A reduced K_d_ value and, hence, a stronger interaction was also detected for the interaction of cTnI-D127Y with cTnT compared to cTnI-WT (cTnI-D127Y K_d_ = 43.80 ± 14.81 nM vs. cTnI-WT K_d_ = 109.67 ± 24.45 nM) (Figure 4D).

The K_d_ value derived from the binding curves of cTnC-G34S with cTnI was unchanged compared to that of cTnC-WT (cTnC-G34S K_d_ = 186.43 ± 15.09 nM vs. cTnC-WT K_d_ = 179.57 ± 47.22 nM) (Figure 4E).

The cTnC-G34S subunit exhibited a decreased binding affinity towards cTnT compared to cTnC-WT (cTnC-G34S K_d_ = 14.28 ± 6.62 µM, vs. cTnC-WT K_d_ = 2.92 ±1.21 µM), suggesting a weaker interaction between cTnC-G34S and cTnT (Figure 4F, Table S2.3).

Thus, the interaction of the mutant troponins with actin was not impaired in contrast to the intramolecular interactions within the troponin complex. This might contribute to an impaired transmission of the Ca^2+^-signal or might even affect the structural integrity of the thin filaments.

### 2.4. Structural Analysis

**Figure 5:**
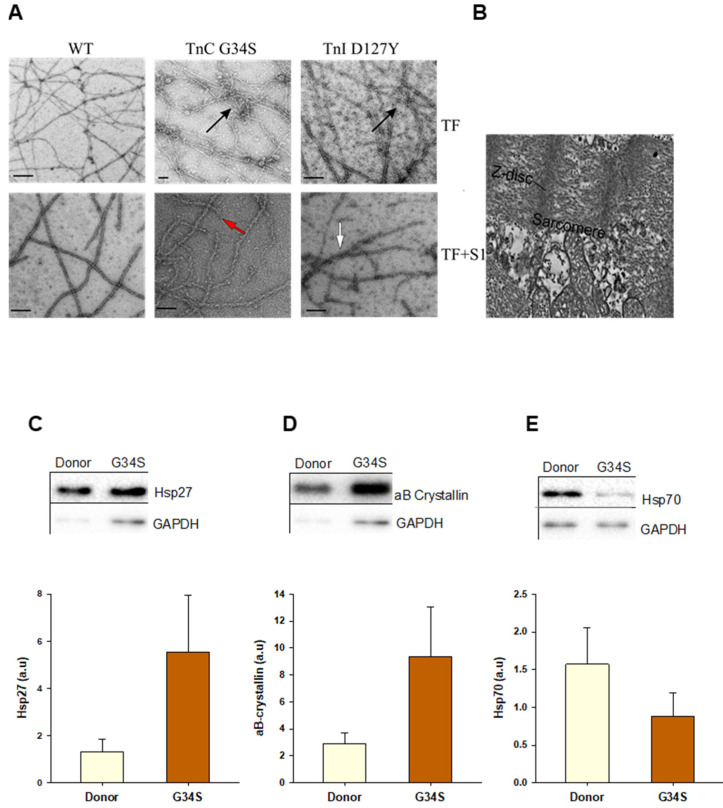
(**A**) Representative electron microscopic images of negatively stained skeletal F-actin decorated with cTm and troponin complexes (cTn-WT, cTn-G34S, or cTn-D127Y) ± myosin sub-fragment S1. The arrows indicate filament fragmentations (red), aggregation (black), and irregular decoration with myosin-S1 (white). (**B**) samples from TnC G34S patient’s frozen cardiac tissue. The bars represent 100 nm. (**C**) Heat shock protein 27 (Hsp 27) protein levels in donor and TnC-G34S tissue samples. (**D**) αβ-crystallin protein levels in donor and TnC-G34S tissue samples (**E**) Hsp 70 protein levels in donor and TnC-G34S tissue samples. Data are shown as mean ± SEM; *n* = 5 samples/group.

The stability and the structural integrity of the mutant thin filaments were analyzed by electron microscopy (EM). EM images of negatively stained skeletal F-actin filaments decorated with cardiac tropomyosin and cTn WT showed a normal linear appearance. EM images of the filaments containing cTn mutants demonstrated a mutation-induced disturbance in the thin filament morphology; they appeared to be partially fragmented and bundled with thin short filamentous structures in between. The additional decoration with myosin sub-fragment S1 resulted in a typical arrowhead appearance (i.e., full decoration) of the wild type filament, whereas the mutant filaments were only partially decorated (Figure 5A).

To compare the architecture in the original heart with reconstituted thin filaments, EM images of tissue samples from the cTnC-G34S patient were recorded (Figure 5B). The tissue samples revealed a clear disruption in the overall sarcomere morphology.

### 2.5. Western Blot Analysis for Proteostasis

The data obtained from the functional and interaction assays show that the contractile function, i.e., thin filament activation, myosin motor kinetics, as well as intramolecular interactions, are impaired rather similarly by troponin complexes containing the mutated subunits of cTn. Furthermore, the structural integrity of thin filaments containing the mutations was disturbed in terms of filament stability and aggregation. Importantly, severe disruption of the sarcomere structure was observed by EM in the G34S myocardial tissue (Figure 5B). Therefore, we aimed to analyze the aggregation phenomena by checking the expression levels of small heat shock proteins (sHsps) in tissue samples from the cTnC-G34S patient using western blot (Figure 5C). sHsps are known to translocate to sarcomeres and prevent stress-induced protein aggregation, thereby stabilizing the cardiomyocyte structure [24,25]. Compared to the donor group, the expression of Hsp27 and αβ crystallin was upregulated (Figure 5C,D), indicating stress-induced alterations in cardiomyocyte proteostasis [26,27], whereas the expression of Hsp70 was slightly reduced (Figure 5E).

**Figure 6:**
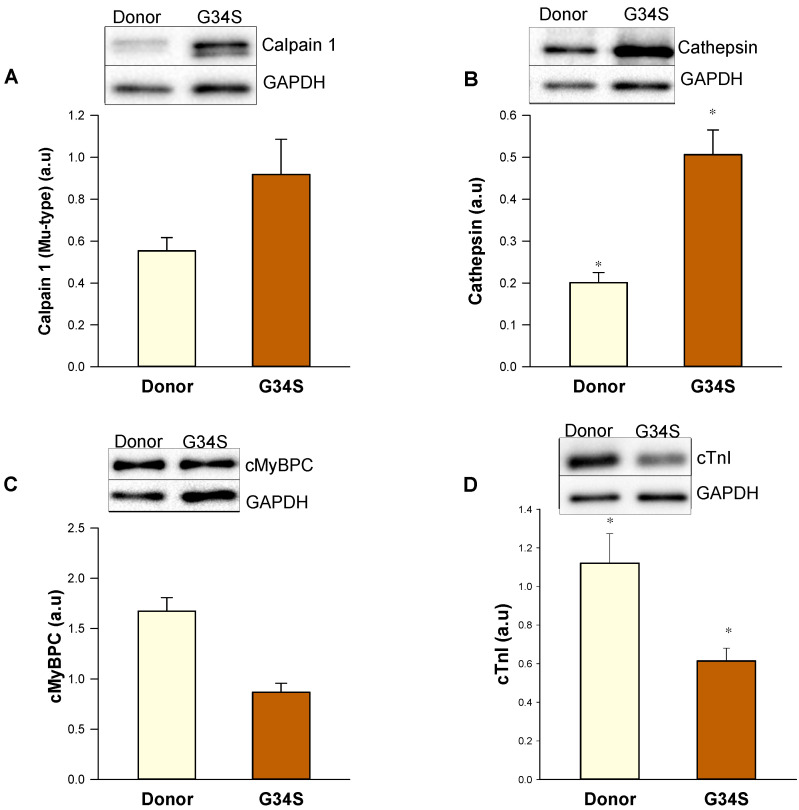
Western blot analysis of components of protein quality control system in the cardiac tissue of the G34S patient. (**A**) Calpain 1 protein levels. (**B**) Cathepsin protein levels. (**C**) cMyBPC protein levels (**D**) cTnI protein levels. Data are shown as mean ± SEM; *n* = 5 samples/group. * *p* < 0.05 controls vs. cTnC G34S.

As sHsps are shown to target misfolded proteins to the proteasome, we aimed to evaluate the proteolytic events by western blot analysis of expression levels of proteins that are involved in proteolysis. Western blot analysis revealed increased expression levels of proteases, such as calpain 1 large subunit and cathepsin L (Figure 6A,B). Furthermore, the protein levels of cMyBPC and cTnI were decreased in the patient’s heart compared to donor tissue (Figure 6C,D), which might be due to the increased proteolysis and protein degradation events in the cTnC-G34S tissue. In addition, cTnI levels can be further decreased due to the early stage of the isoform transition from embryonic slow skeletal TnI to cTnI, which occurs during the first year of life [28].

### 2.6. The Effects of Troponin Targeting Agents on Troponin Mutations

In our study, we showed that both mutants severely affected the function, Ca^2+^-sensitivity and structural stability. Therefore, we wondered whether drugs, such as the green tea catechin(-)-epigallocatechin-3-gallate (EGCg, Ca^2+^-desensitizer) and levosimendan (Ca^2+^-sensitizer), could influence the parameters investigated above.

**Figure 7:**
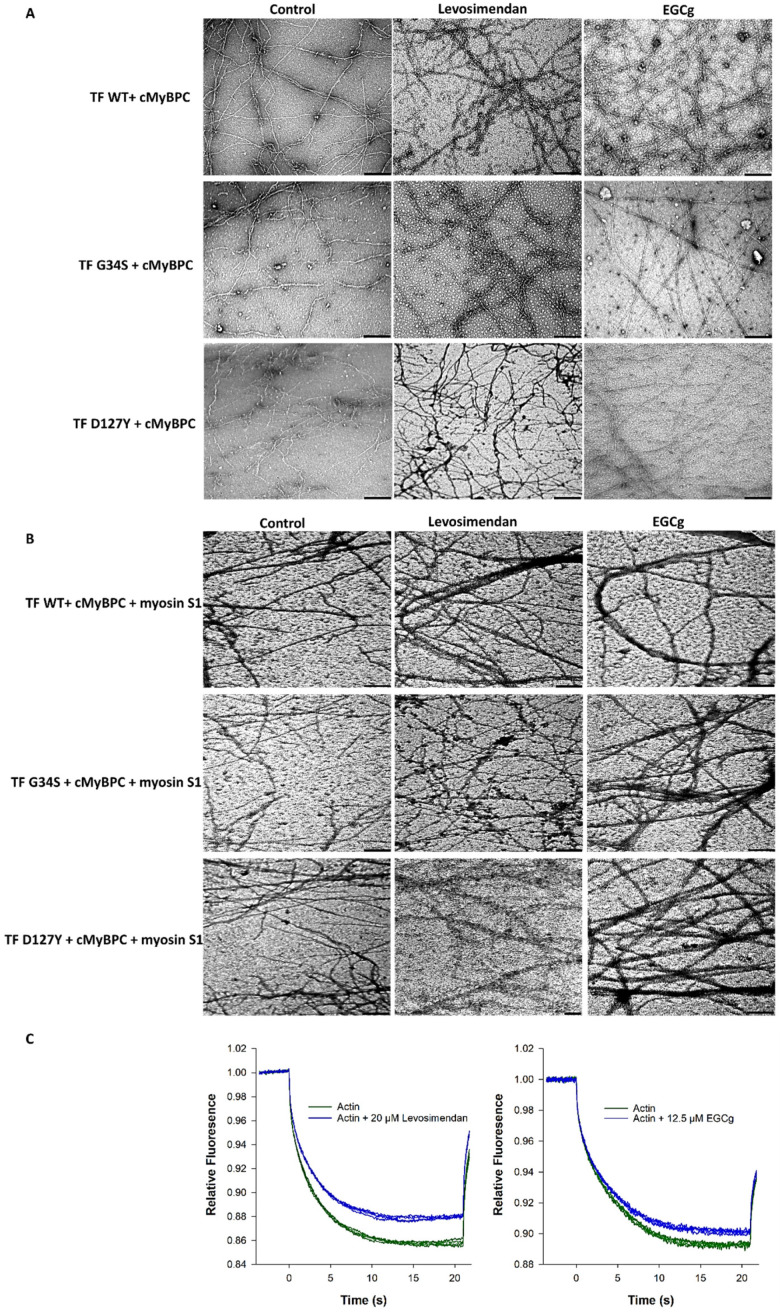
(**A**) Representative electron microscopic images of negatively stained skeletal F-actin reconstituted with cTm, cMyBPC C0-C2, and troponin complexes (cTn-WT, cTn-G34S, cTn-D127Y) in the presence of either 20 µM EGCg or 20 µM levosimendan and an untreated control group. The bars represent 250 nm in all images. (**B**) After decoration with myosin-S1. (**C**) Direct interaction study of fluorescently labeled G-actin with either 20 µM levosimendan (left) or 12.5 µM EGCG (right) measured by MST (binding check experiment). The relative fluorescence was plotted vs. time (s), (*n* = 4).

To determine the effects of troponin targeting agents (EGCg and levosimendan) on the structure and morphology of thin filaments, EM images of negatively stained skeletal F-actin (reconstituted with cTn, Tm, cMyBPC C0-C2, myosin-S1 at 6:1:1:1:1 ratio, respectively) were recorded in the presence of either 20 µM EGCg or 20 µM levosimendan (Figure 7A,B). The EM images of wild type filaments treated with 20 µM EGCg or 20 µM levosimendan revealed apparent variations, such as increased aggregation and elongation, compared to controls; similar effects were observed in thin filaments containing cTnC-G34S and cTnI-D127Y. Interestingly, both EGCg and levosimendan seemed to improve the aberrant structure in terms of reducing the fragmentation and improving filament linearity. These effects were more prominent with EGCg than with levosimendan. Furthermore, with levosimendan, filament bundling was increased (Figure 7A). Decoration with myosin-S1 was nearly unchanged in the treated wild type filaments compared to controls (Figure 7B). EGCg improved myosin-S1 decoration in both TF-G34S and TF-D127Y; however, only partial myosin-S1 decoration was observed in both TF-G34S and TF-D127Y after levosimendan treatment (Figure 7B).

As EM records of thin filaments treated with levosimendan or EGCg showed strong morphological alterations compared to controls, we investigated the interaction between labeled actin monomers and these compounds using MST. The binding check experiment revealed a direct interaction between labeled actin with 12.5 µM EGCg and with 20 µM levosimendan. As shown in Figure 7C, both 12.5 µM EGCg and 20 µM levosimendan caused binding-induced changes in the thermophoretic behavior of actin, which was detected with a relatively high signal-to-noise ratio.

**Figure 8:**
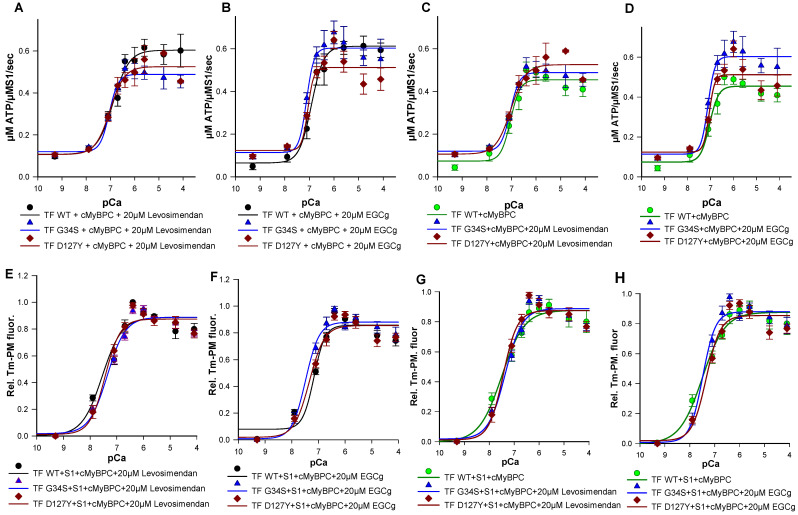
(**A**) Ca^2+^-dependent stimulation of the acto-myosin-S1-ATPase of thin filaments (TF) reconstituted with troponin complexes (Tn-WT, Tn-G34S or Tn-D127Y), skeletal actin, tropomyosin, and cMyBPC C0-C2 in the presence of 20 µM levosimendan (**B**) In the presence of 20 µM EGCg. (**C**) Levosimendan treated TF-G34S and TF-D127Y vs. untreated TF-WT. (**D**) EGCg treated TF-G34S and TF-D127Y vs. untreated TF-WT. The data points represent the rate of ATP hydrolysis by myosin-S1 as mean ± SEM vs. pCa and are fitted to Hill equation to obtain pCa_50_ and nHill values (*n* = 5–8). (**E**) Representative data for the Ca^2+^-dependent increase in fluorescence intensity of thin filaments reconstituted with troponin complexes (Tn-WT, Tn-G34S or Tn-D127Y), skeletal actin, pyrene maleimide-labeled tropomyosin, cMyBPC C0-C2, and myosin-S1 in the presence of 20 µM levosimendan. (**F**) In the presence of 20µM EGCg. (**G**) Levosimendan treated TF-G34S and TF-D127Y vs. untreated TF-WT (**H**) EGCg treated TF-G34S and TF-D127Y vs. untreated TF-WT. Data points are presented as mean of normalized fluorescence ± SEM vs. pCa and fitted to Hill equation to obtain the pCa_50_ and nHill values (*n* = 6–9).

We then analyzed the effects of troponin targeting agents on the ATPase activity of wild type and mutant filaments. While no significant changes were detected in Ca^2+^-sensitivity or co-operativity (Table S2.1), addition of 20 µM levosimendan resulted in a significant increase in the maximal ATPase activity of wild type filaments (TF-WT Δy_max_ = +0.149) (Table 2), which was consistent with higher levels of force generation previously reported upon levosimendan treatment. A significant decrease in the maximal ATPase activity was observed for the TF containing cTnI-D127Y (TF-D127Y) (Δy_max_ = −0.011) compared to their respective untreated filaments. The same tendency was obtained with TF containing cTnC-G34S (TF-G34S) (Table 2, Figure 8A,C). The decrease in the maximal actomyosin-S1-ATPase activity of mutant filaments in the presence of levosimendan was highly significant when compared to treated wild type filaments (TF-G34S Δy_max_ = −0.115, TF-D127Y Δy_max_ = −0.078) (Table 2, Figure 8C). Furthermore, no significant effects on the y_min_ or activation amplitudes were detected for all troponin variants (Table 2).

The treatment with 20 µM EGCg resulted in a significant increase in maximal myosin-S1-ATPase activity stimulation of both TF-WT and TF-G34S, but not of TF-D127Y, when compared to their untreated respective filaments (TF-WT Δy_max_ = +0.157, TF-G34S Δy_max_= +0.086).

When compared to treated wild type filaments, EGCg decreased the maximal ATPase activity of TF-D127Y but not TF-G34S (TF D127Y Δy_max_ = −0.1) (Table 4). y_min_ was found to be slightly decreased in treated TF-WT and increased in treated TF-G34S and TF-D127Y compared to their respective non-treated filaments; however, the changes in y_min_ were non-significant. The activation amplitude of TF-D127Y was significantly decreased compared to the treated wild type filaments but unchanged for all thin filament constructs when compared to their respective untreated ones (Table 2, Figure 8B,D). By trend for cTnI D127Y mutation, both levosimendan and, to an even greater extent, EGCg seemed to shift ymax closer to untreated WT levels corresponding to the healthy normal state (Table 2). In contrast, for cTnC-G34S, EGCg seemed to have an adverse effect on ymax, while levosimendan still restored it to untreated WT levels, though differences were not significant.

**Figure 9:**
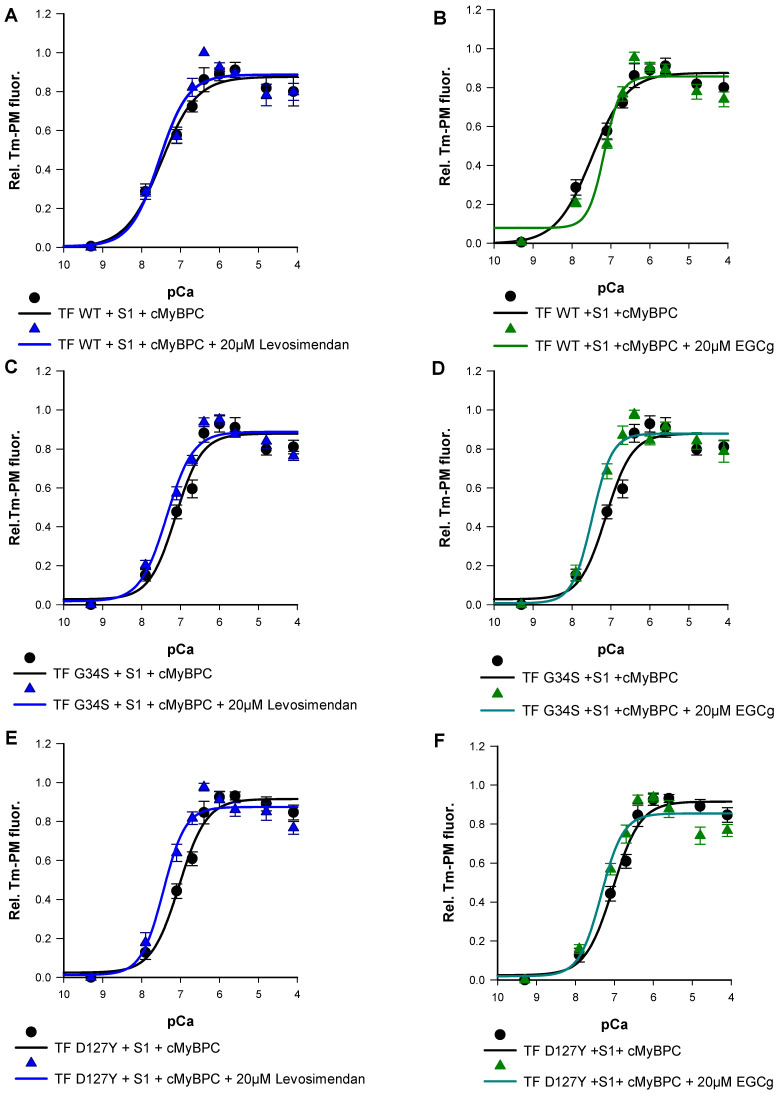
Representative data for the Ca^2+^-dependent increase in fluorescence intensity of thin filaments in the presence and absence of 20 µM levosimendan, which were reconstituted with skeletal actin, pyrene-maleimide-labeled tropomyosin, cMyBPC C0-C2, myosin-S1 and (**A**) cTn-WT, (**C**) Tn-G34S, (**E**) Tn-D127Y. (**B**) Representative data for the Ca^2+^-dependent increase in fluorescence intensity of thin filaments in the presence and absence of 20 µM EGCg, which were reconstituted with skeletal actin, Pyrene maleimide-labeled tropomyosin, cMyBPC C0-C2, myosin-S1, and cTn-WT, (**D**) Tn-G34S, (**F**) Tn-D127Y. Data points represented as mean of normalized fluorescence ± SEM vs. pCa and fitted to Hill equation to obtain pCa_50_ and nHill values (*n* = 6–9).

Finally, we studied the effects of EGCg and levosimendan on Ca^2+^-triggered activation of thin filaments reconstituted with troponin mutations by monitoring Tm-PM fluorescence. Previous studies have shown the EC50 of levosimendan in force-pCa relations on skeletal muscle fibers to be 15–20 µM [29]. To determine the effective dose in our assay, levosimendan was added to the assay in a concentration series of 10 µM, 15 µM, 20 µM, 25 µM, and 30 µM. Treatment with 20 µM levosimendan significantly increased pCa_50_ values of the mutant filaments compared to their respective untreated ones (Figure 8E,G and Figure 9A,C,E) (TF-G34S + levosimendan ΔpCa_50_ = +0.23, TF-D127Y + levosimendan ΔpCa_50_ = +0.40). However, the wild type filaments appeared to be less affected by levosimendan (TF-WT + levosimendan ΔpCa_50_ = +0.06). No significant effects on the co-operativity were observed (Table 3).

EGCg has been demonstrated to decrease Ca^2+^-sensitivity in myofilaments [20,30]. In order to investigate the dose-dependent effects of EGCg on myofilament activation, varying concentrations of EGCg were used (10 µM, 15 µM, 20 µM, 25 µM, and 30 µM). As expected, we found significantly reduced pCa_50_ of the wild type filaments at a concentration of 20 µM (TF-WT + EGCg ΔpCa_50_= −0.33) (Figure 8F,H and Figure 9B,D,F). A higher value of _n_Hill was detected, indicating an increased co-operativity of thin filament activation; however, the increase was not statistically significant (Table 3) (Figure 8 and Figure 9).

Surprisingly, treatment with 20 µM EGCg has significantly increased pCa_50_ of the mutant filaments compared to their respective untreated ones (TF-G34S + EGCg ΔpCa_50_ = +0.36, TF-D127Y + EGCg pCa_50_ = +0.30). No significant effects on the thin filament co-operativity were observed (Figure 8 and Figure 9).

We summarized our main findings in Table 5. Both mutants, either leading to NCM or RCM, impair contractile performance and increase significantly the Ca^2+^-responsiveness on myofiber or cardiomyocyte level. On a reconstituted myofilament level, the Ca^2+^-sensitivity was either marginally increased (acto-myosin-ATPase activity) or even decreased (thin filament activation). Maximal tension was reduced with cTnC-G34S.

In the heart tissue of the cTnC-G34S patient, the protein quality control system seemed to be impaired, as reflected by increased levels of sHsp27 and αβ-crystalline, reduced levels of Hsp70, and increased levels of proteases. Due to tissue unavailability, the same analysis for cTnI-D127Y could not be performed.

Intramolecular interactions within the troponin complex are disturbed showing stronger affinities between the troponin subunits when cTnI-D127Y was present, as well as a weakened interaction of cTnC-G34S with cTnT.

The structural integrity of sarcomers in case of cTnC-G34S and of reconstituted mutant thin filaments with cTnC-G34S or cTnI-D127Y was severely disrupted, showing fragmented, shorter, and bundled filaments and diminished decoration with myosin-S1.

Both cTn targeting drugs (levosimendan and EGCg) increased the maximal acto-myosin-S1-ATPase activity in vitro for wild type but not mutant filaments. The Ca^2+^-sensitivity of mutant thin filament activation was increased.

Both drugs interact directly with actin and lead to less fragmented and longer mutant thin filaments. However, bundling was increased, especially under the influence of levosimendan, and decoration with myosin-S1 was restored to normal with ECGg.

## 3. Discussion

Here, we report the functional analysis of two cardiac troponin variants causing severe infantile cardiomyopathy. One *missense* mutation occurred in TNNC1 (p.cTnC-G34S) and one in TNNI3 (p.cTnI-D127Y). cTnC-G34S was identified in a newborn child. The attributed pathological consequences were a cardiomyopathy characterized by an enlarged and fibrotic heart and sponge-like ventricles, leading to end-stage heart failure with a non-compaction phenotype. The life of this child was saved by heart transplantation at the age of 10 weeks. The parents gave informed consent to use the explanted, diseased heart for further scientific analysis. cTnI-D127Y was diagnosed in an 8-month-old child with signs of heart failure due to restrictive physiology of both ventricles. VAD treatment was unsuccessful, and the child died at the age of 15 months. No consent was given to use the heart of the deceased child for further scientific evaluation.

In both cases, family analysis revealed that both the mutations are de novo and were classified as likely pathogenic according to the American College of Medical Genetics and Genomic (ACMG) guidelines. We first investigated the consequences of cTnC-G34S and cTnI- D127Y on the performance of skinned cardiomyocytes, or skinned fibers and reconstituted thin filaments. Subsequently, intramolecular interactions and interactions of the respective troponin complexes with actin, as well as the structural integrity of reconstituted thin filaments, were investigated. The three state model as proposed by Mc Killop and Geeves [31] forms the frame, to which the functional alterations of the troponin subunit mutations have to be related.

Functional mechanical measurements were performed with skinned cardiomyocytes isolated from the explanted diseased heart in case of the *TNNC1*-p.cTnC-G34S mutation. The data obtained showed an increase in the Ca^2+^-sensitivity but a force reduction at activating Ca^2+^-concentrations compared to a donor heart, typical for heart failure [32,33]. An increase in Ca^2+^-sensitivity is uncommon in systolic diseases as DCM or NCM in adults, but Nakano et al., in 2019, showed that end stage pediatric DCM is characterized by an increased Ca^2+^-sensitivity [34], in contrast to adult DCM. In addition, previous analyses of mutations of this cTnC region (Ca^2+^ binding site I) have reported similar alterations but were classified as HCM. Near position 34 in cTnC, the missense mutations L29Q and A31S have been described [35,36,37]. These amino acid replacements, including G34S, reside in the loop of the Ca^2+^ binding site I of cardiac TnC, which is non-functional due to missing Ca^2+^ coordination sites. The L29Q variant, which was first detected in a 60-year-old patient [36] has been described to alter the kinetics of cross-bridge cycling via altered Ca^2+^-affinity, though conflicting results have been reported concerning its Ca^2+^-affinity (an increase, a decrease or no effect) [38]. Furthermore, it blunts the effects of PKA-dependent phosphorylation of cTnI [39]. The A31S variant has clearly been shown to increase the Ca^2+^-sensitivity and, as L29Q, blunts effects of PKA-dependent phosphorylation on contraction [37]. A genetic screening of a children cohort revealed that the A31S mutation occurred as a de novo mutation with severe disease progression and the need of transplantation at the mean age of 7 years [35].

The increase in the Ca^2+^-sensitivity in cTnC-G34S cardiomyocytes, as measured here, might be smaller in vivo since, for comparison, we used donor tissues from adult hearts due to the unavailability of an appropriate infant donor heart. It has been described that the Ca^2+^-sensitivity in the newborns is higher than in adult hearts due to the troponin T isoform exchange during development [40]. In addition, the predominant expression of ssTnI instead of cTnI in newborns contributes to an increased Ca ^2+^-sensitivity. compared to adults expressing cTnI only [28].

The elevation of the Ca^2+^-sensitivity may be due to a higher Ca^2+^-binding-affinity as measured by an increase in fluorescence using thin filaments decorated with cTn containing IAANS labeled cTnC-G34S in comparison to wild type filaments (see supplements). It appears, however, unlikely that the serine residue provides a coordination site for Ca^2+^ within the non-functional Ca^2+^-binding loop because it is not located at one of the coordination sites (sequence position 31 and 29). However, this mutation could be a gain of function mutation by altering the dynamics of the N-terminal lobe of cTnC, thereby increasing the affinity of the Ca^2+^-binding to site II or favoring the open (fully activated) state, as proposed for several HCM mutations [41]. In addition, there is evidence of a direct interaction between cTnT and cTnC N-terminal lobe, which restricts dynamics of cTnC [42]. In case of the G34S mutant, we observed decreased binding affinity between cTnC and cTnT, which could lead to increased activation by Ca^2+^.

Similar experiments concerning the TNNI3-p.cTnI-D127Y variant were only possible after exchanging endogenous cTnI with cTnI-D127Y in skinned guinea pig fibers. The data obtained show a clear increase in the Ca^2+^-sensitivity with cTnI-D127Y containing fibers. The force measurements, however, showed large variations at activating Ca^2+^, though a considerable number of different experiments was performed. Therefore, no definite statement can be made about the effect of cTnI-D127Y on the maximal force, though, by trend, a reduction can also be assumed. Generally, for cTnI RCM variants, a largely increased Ca^2+^-sensitivity has been described [2], as recently demonstrated with other infantile cTnI-RCM variants, TNNI3-p.cTnI-R170G and -R170W [22].

Next, we integrated both purified mutant proteins in cTn complexes, which were subsequently used to decorate F-actin, together with cTm, to reconstitute functional thin filaments. Using such reconstituted thin filaments (TF), we determined the Ca^2+^-dependency of their ability to stimulate the myosin-S1 ATPase activity and to induce the movement of pyrene-labeled cTm from the blocked to activated states (as measured by the increase in pyrene-fluorescence). In contrast to the muscle fiber data, we observed for both mutant TFs only a small, but not significant, increase in the Ca^2+^-sensitivity of the myosin-S1 ATPase stimulation and a significantly increased maximal ATPase activity.

The apparent discrepancies between cardiomyocytes, fibers, and reconstituted TFs could be explained by the different organization levels of the model systems: In intact cardiomyocytes, direct or indirect interactions with other sarcomeric proteins or post-translational modifications might modify the response of cTnC to alterations of the Ca^2+^-concentration and, thereby, contribute to the observed effects, as well as additional variations in other genes, as detected in the G34S and D127Y patients, or the presence of troponin subunits in the nuclei [12]. The advantage of the reconstituted system is, however, the fact that the functional alterations caused by a mutation can be detected without possible compensating effects initiated in explanted cardiomyocytes by the highly complex sarcomeric organization or imposed by previous medical treatments.

In addition, we performed an analysis of the Ca^2+^-dependency of the pyrene-cTm movement. We observed no change by the mutants in comparison to WT-cTn. Addition of myosin-S1, however, led to a clear increase for WT-cTn but to significantly reduced Ca^2+^-sensitivity for the TFs containing either mutant, indicating that more Ca^2+^ is needed for the mutant cTn containing TFs to shift the tropomyosin towards the open state in the presence of myosin-S1. The Hill coefficients were not altered significantly, indicating only small changes in co-operativity which might impair the highly co-operative movement of Tm. Furthermore, the apparent ability of the cTnC and cTnI mutants to fragment TFs as shown by EM is likely to affect the co-operativity of the TM movement of the actin filaments.

In summary, both cTnC-G34S and cTnI-D127Y induced a clear dysregulation of contractile function. As we demonstrated previously for other variants [22], this finding was accompanied by an altered interplay of sarcomeric proteins and a disrupted structural integrity of reconstituted thin filaments. Both mutants investigated did incorporate normally into reconstituted thin filaments, and no significant effect on binding to actin was obtained.

However, the intramolecular interactions were altered differently by the two mutants. Thus, cTnI-D127Y showed a significantly increased binding affinity towards cTnC and cTnT, which might indicate an increased rigidity and, thus, reduced dynamics of the troponin complex. In contrast, cTnC-G34S showed a decreased binding affinity towards cTnT, indicating a reduced stability of the troponin complex. All these alterations in the interactions within the Tn complex interactions might point to an impaired transfer of the Ca^2+^ signal to the other components of the thin filament and, in case of the RCM variant TNNI3-p.cTnID127Y, might contribute to the myocardial stiffness, a hallmark for RCM. In addition, disturbed protein–protein interactions might also affect the structural integrity, stability, and dynamics of the thin filaments. Indeed, the EM analysis of the p.cTnC-G34S tissue (Figure 5) showed largely disrupted sarcomeres.

Consistently, the reconstituted thin filaments also exhibited an abnormal appearance in the EM images. A significant increase in filament breaks and filament clustering indicating aggregation was observed, not only when TFs contained cTnC-G34S but also cTnI-D127Y. Similar disturbances have been described by our group previously with the infantile RCM variants p.cTnI-R170G/W [22]. Furthermore, here, we observed a reduced decoration with myosin-S1, which would match the reduced maximal force production clearly seen with the p.cTnC-G34S variant in skinned cardiomyocytes. Destabilization of thin filament structure can also lead to mechanical unloading of the sarcomers, which would also explain the force reduction observed without reduction in the myosin-S1 ATPase kinetics.

Due to the bundling of actin filaments and massively disrupted sarcomeres, we used the explanted heart with the p.cTnC-G34S variant to investigate by Western blotting signs of aggregation and the presence of proteolytic markers. The reduction in Hsp70 in the patients tissue indicates that the cardiomyocytes might be less protected against cell death, as has been proposed by Latchman, in 2001 [43], and protein aggregation [44].

The small heat shock protein Hsp27 and αβ-crystallin are thought to inhibit aggregation, as shown several times for neurodegenerative diseases [45]. Moreover, sHsps are shown to translocate to sarcomeres and prevent stress-induced protein aggregation, thereby stabilizing the cardiomyocyte structure [24,46]. Both Hsp27 and αβ-crystallin co-localize with titin at the Z-disc and I-band titin, thereby protecting against Ig domain unfolding, aggregation, and subsequent high myocyte stiffness. However, previous studies by us and others reported the altered localization of sHsps away from the Z-disc and I-band in HCM tissue [25], an effect that was attributed to oxidative-stress induced alterations in Hsps, such as S-glutathionylation and hypophosphorylation. Hence, despite the elevated expression levels, sHsps seemed to fail in exerting their cytoprotective role, and they could not maintain a balanced proteostasis and protect against protein aggregation [25]. Consistent with these studies, we detected high levels of αβ-crystallin in cTnC-G34S patient’s cardiac tissue, as well as protein aggregation, which, according to our observation of thin filament bundling, might be caused by the cTn mutants.

The increased levels of the proteases calpain-1 and cathepsin are strong indicators for increased protein degradation. Accordingly, immunohistologic analysis of the G34S myocardial tissue showed decreased levels of myofilament proteins. Our findings imply that the protein control system is impaired, which might contribute to the severe alterations in the structural integrity of the thin filaments and cardiomyocytes we observed here. There are not many studies available investigating the protein control system in genetically-based cardiomyopathies as NCM and RCM. Kumarapeli et al., in 2010 [47], for example showed that deficiency of αβ crystallin in mice in combination with chronic pressure overload led to restrictive phenotype. In contrast, immunohistology in biopsy samples of the cTnI- D127Y patient (Figure S1.4) did not reveal a reduced expression of myofilament proteins.

Finally, we tested whether we could restore the contractile function and thin filament structure by using cTn targeting drugs, such as levosimendan, a pyridazinondinitrile derivative, or EGCg, a polyphenol. Levosimendan is known to interact directly with cTnC, thereby increasing its Ca^2+^-affinity and Ca^2+^-sensitivity of the contractile machinery [29]. Furthermore, levosimendan showed other beneficial effects as vasodilator, as an effector of mitochondrial ATP production and as PDE (phosphodiesterase) inhibitor [48]. Levosimendan exerts inotropic and lusitropic effects in humans [49] and is applied to heart failure patients with reduced systolic function to increase contractility. Thus, it was also used in the therapy of the G34S patient. In addition, EGCg has multiple effects on the cardiovascular system, besides decreasing the Ca^2+^ binding affinity to cTnC by directly binding to cTnC, as being anti-fibrotic, anti-apoptotic, anti-oxidant, and anti-hypertrophic, thereby affecting several signaling pathways [30,50]. Here, we show, for the first time, that both drugs bind to actin and elongate actin filaments, thereby reducing small thin filament fragments. However, bundling of thin filaments was not reversed and was even augmented by levosimendan. Furthermore, both drugs could not completely restore the function of reconstituted thin filaments, there was no effect on the Ca^2+^-dependency of the acto-myosin-S1- ATPase activity, but thin filament activation was restored, which was more prominent with EGCg. Since the cardiovascular effects of both drugs are promoted via multiple mechanisms rather than single drug-receptor interaction [48], the results obtained in this study might be dependent on the organization levels of the model system. Hence, both drugs seem to act beneficial in some structural and functional aspects; however, this must be investigated in more detail in future.

## 4. Materials and Methods

All materials were purchased from Sigma Aldrich, St. Louis, MO, USA, except where otherwise indicated.

### 4.1. Genetic Analyses

Molecular genetics were performed after oral and written informed consent. The study protocol was approved by the local ethics committee. DNA isolation and panel sequencing were performed as described previously [51,52]. Briefly, DNA was isolated from blood with the High Pure PCR Template Preparation Kit^®^ (Roche Diagnostics GmbH, Mannheim, Germany) and used for cardiac gene enrichment re-sequencing on a MiSeq^®^ next-generation sequencing system according to the manufacturer’s instructions (TruSight^TM^ Rapid Capture Preparation Kit; Illumina, San Diego, CA, USA). The patients were screened for variants in 174 genes associated with inherited cardiac conditions using the TruSight^TM^ Cardio gene panel (Illumina). For variant annotation, Studio^TM^ v3.0 (Illumina) was used. Significant variants were verified by Sanger sequencing using the BigDye^®^ Terminator v1.1 Cycle Sequencing Kit on an ABI PRISM^®^ 3500 genetic analyzer (Applied Biosystems, Foster City, CA, USA). Variant classification was performed according to the guidelines of the American College for Medical Genetics and Genomics (ACMG), and databases Clinvar, Human Gene Mutation Database (HGMD) were used. Minor allele frequencies were checked in GnomAD [53] (Figures S1.2 and S1.3, Tables S1.1 and S1.2).

### 4.2. Human Heart Tissues

LV tissue from non-failing donor hearts (*n* = 10; male; average age 40 years) served as reference, and non-failing cardiac LV tissue was obtained from donor hearts for which no suitable transplant recipient was found. The donors had no history of cardiac disease, a normal ECG and normal ventricular function on echocardiography performed within 24 h prior to heart transplantation. Tissues were collected in cardioplegic solution and stored in liquid nitrogen until use. For studies including human donor tissues, samples were obtained after informed consent and with approval of the local Ethics Committee (St Vincent’s Hospital of Sydney, Sydney, Australia, Human Research Ethics Committee; File number: H03/118; Title: Molecular Analysis of Human Heart Failure). The investigation conforms to the principles outlined in the Declaration of Helsinki. For cTnC-G34S and cTnI-D127Y, the study conformed with the declaration of Helsinki and was approved by the ethics committee of the Ruhr-University Bochum, located in Bad Oeynhausen (Vote No. 2018-367).

### 4.3. Proteins

#### 4.3.1. Site Directed Mutagenesis

The human cardiac troponin C mutant (p.cTnC-G34S) was constructed from its respective wild type pET3c plasmid by using the Expand Long Range kit (Roche, Switzerland), and the primers forward: 5’-GGC GCT GAG GAT AGC TGC ATC AGC A-3’, and reverse: 5’-TGC TGA TGC AGC TAT CCT CAG CGC C-3’. For the introduction of the p.D127Y mutation in human cTnI (pET3c-TTNI3), the following primers were used: forward 5′-GAG ATT GCA TAT CTG ACT CAG AAG ATC TTT GAC CTT CG-3′, reverse 5′-CTG AGT CAG ATA TGC AAT CTC CGT GAT GTT CTT GGT G-3′. The correct sequence of the constructs was confirmed at LGC Genomics (Berlin, Germany).

#### 4.3.2. Protein Expression and Purification

For protein expression, the plasmids encoding human cTnC WT, cTnC G34S, and cMyBPC C0-C2 (in pET28a) were transformed into *E.coli* T7 Express lysY cells, while the plasmids encoding cTnI WT, cTnI D127Y, and cTnT WT were transformed into *E. coli* BL21 (DE3).

The isolation of cTnC WT/G34S was performed following the method described by Babu et al. (1992) [54]. cTnI WT/D127Y were purified according to Reiffert et al. (1999) [55]. cTnT WT was isolated according to Deng et al. (2001) [56]. For the reconstitution of human cardiac wild type (cTn-WT) and mutant (cTn-G34S and cTn-D127Y) troponin complexes, the individual subunits were mixed at an equimolar ratio as previously described [56].

The cMyBPC C0-C2 fragment comprising amino acids 1-452, expressed with an N-terminal His_6_-tag, was purified using Ni^2+^-magnetic beads (SERVA Ni-NTA Magnetic Beads, Heidelberg-Germany) according to manufacturer’s instructions [22].

Monomeric G-actin was isolated by standard methods from acetone dried powder prepared from rabbit skeletal muscles [57]. Tropomyosin was chromatographically purified from acetone dried powder prepared from pig heart according to Bailey et al. [58]. Myosin was isolated from fresh pig heart ventricles. Myosin sub-fragment S1 was prepared from full length myosin by enzymatic digestion using α-chymotrypsin, as well as further purified on DEAE-cellulose [59,60]. The protein purity was controlled by SDS-PAGE (Figures S2.1 and S2.2).

### 4.4. Functional Assays

#### 4.4.1. Preparation, Troponin Exchange, and Force Recordings in Skinned Fibers

Skinned cardiac fibers were prepared from guinea pigs. The endogenous troponin was exchanged by the recombinant human troponin complex containing cTnI D127Y or wild type cTnI in an exchange buffer containing 10 mM Tris, 132 mM NaCl, 5 mM KCl, 1 mM MgCl_2_, 5 mM EGTA, 1 mM NaN_3_, 5 mM DTT, 0.5 mM (4-(2-aminoethyl)-benzene sulfonyl fluoride (AEBSF), 15 μM antipain, 0.8 μM aprotinin, and 10 μM leupeptin (pH 7.0) [22]. The skinned fiber bundles were mounted horizontally between two clamps connected to a length driver and the tip of the force transducer. For the exchange, the fibers were incubated at RT for 3 h in the exchange buffer containing 3 mg/mL human recombinant troponin complex. The extent of troponin-exchange was quantified by SDS-PAGE (12.5% gels) and analysis of Coomassie stained gels using Phoretics (Figure S3). Maximal Ca^2+^-activated force and Ca^2+^-sensitivity of isometric tension were examined by exposing the skinned fibers to activating solutions with free Ca^2+^-concentrations ranging from pCa 7.0 to 4.7, until a plateau in force was reached.

#### 4.4.2. Cardiomyocyte Isolation and Force Recordings

Single cardiomyocytes were isolated from a frozen tissue sample of TnC G34S patient’s and human donor hearts. Briefly, the tissue samples were defrosted in a relaxing solution (1 mM free Mg^2+^, 4 mM Mg-ATP, 2.0 mM EGTA, 100 mM KCl, 10mM imidazole, pH 7.0). Cardiomyocytes were enzymatically isolated, and then mechanically disrupted and incubated for 5 min in relaxing solution containing 0.5% Triton X-100 to remove all membrane structures, as described in detail by Hamdani et al. (2013) [61]. The cell suspension was washed 5 times in relaxing solution. Skinned human cardiomyocytes were then attached between a piezo-electric motor and a force transducer (1600A; with force transducer 403A; Aurora Scientific, Aurora, Ontario, Canada). Force-pCa relations were generated at a pCa range from 9.0 (relaxation solution) to 4.5 (maximal activation). All force values were normalized for cardiomyocyte cross-sectional area. On transfer of the cardiomyocyte from relaxing to activating solution, isometric force started to develop, and steady-state and active force were measured as described previously [62].

#### 4.4.3. Ca^2+^-Dependent Activation of thin Filaments Containing Pyrene-Maleimide-Labeled Tropomyosin

Effects of the troponin mutations on thin filament activation were studied by monitoring the Ca^2+^-dependent excimer fluorescence of pyrene labeled tropomyosin (Tm-PM). The labeling procedure was performed under denaturing conditions [63]. Thin filaments (TF-WT, TF-G34S, and TF-D127Y) were reconstituted with the molar ratio (6:1:1) of 6 µM actin, 1 µM troponin, and 1 µM Tm-PM, respectively, followed by a dialysis step against 3 × 1 L of filament buffer (20 mM HEPES, 70 mM KCl, 5 mM MgCl_2_, 0.5 mM EGTA, 2 mM DTT, pH 7.5) and a subsequent 1:4 dilution step with filament buffer. The activation of the thin filaments was induced by increasing amounts of free Ca^2+^ in the presence of ATP. The transition to myosin bound active state was measured by adding myosin-S1 and/or the N-terminal cardiac myosin binding protein C fragment MyBPC C0-C2 to the assay mix (1:1:6 ratio, myosin-S1 and MyBPC C0-C2 to actin, respectively). The final concentrations of the proteins in the assay were 0.36 μM actin, 0.06 μM troponin, 0.06 μM Tm-PM, 0.06 µM myosin-S1, and 0.06 µM MyBPC C0-C2. The fluorescence intensity was measured in black 96-well microplates at 20 °C using a Tecan infinite 200 microplate reader. The excitation and emission wavelengths used were 340 and 480 nm, respectively. The fluorescence intensities were normalized to F_max_ =1, F_min_ =0, and a non-linear regression was calculated using the Hill-equation:

$$F_{norm}\left(pCa\right)=\frac{1}{\left(1+10^{nH\cdot \left(pCa_{50}-pCa\right)}\right)}.$$

F_norm_: The normalized fluorescence intensity; F_min_: The minimum fluorescence intensity; pCa_50_: The pCa at half-maximum fluorescence intensity; nH: Hill Coefficient.

#### 4.4.4. Ca^2+^-Dependent Acto-Myosin-S1-ATPase Activity Assay

The Ca^2+^-dependent acto-myosin-S1 ATPase activity of reconstituted thin filaments was analysed by an NADH enzyme-linked assay, in which the regeneration of hydrolyzed ATP is coupled to the oxidation of NADH [64]. In this assay, ADP produced by myosin-S1 is re-phosphorylated by phosphoenolpyruvate (PEP) catalyzed by pyruvate kinase (PK) generating pyruvate. Subsequently, the pyruvate is converted by lactate dehydrogenase (LDH) to lactate, and this is coupled with NADH oxidation to NAD^+^. The rate of NADH absorbance decrease can be measured at 340 nm, which is equivalent to the ATP hydrolysis rate by myosin-S1.

The assay was performed at 25 °C (Uvikon 933 Kontron Instruments) to determine the myosin-S1 ATPase stimulation by various reconstituted thin filaments. Thin filaments (TF-Tn-WT, TF-Tn-G34S, and TF-Tn-D127Y) were reconstituted with the molar ratio (6:1:1:1) of 6 µM actin, 1 µM troponin, 1 µM Tm-PM, 1 µM MyBPC C0-C2, respectively, followed by dialysis against 3 × 1 L filament buffer (20 mM HEPES, 70 mM KCl, 5 mM MgCl_2_, 0.5 mM EGTA, 2 mM DTT, pH 7.5) and a 1:4 dilution step with filament buffer. Twenty microliters of thin filaments was added to 140 µL double distilled water (ddH_2_O) supplemented with the regeneration system (2 mM phosphoenolpyruvate, 0.3 mM NADH, 1 mM ATP, and a mixture of pyruvate-kinase and lactate-dehydrogenase resulting in an activity between 50 and 100 units each according to the to the manufacturer’s data (Sigma)) in a final assay volume of 250 µL. EGCg or levosimendan were added to the reaction mix to a final concentration of 20 µM each. The reactions were started by the addition of 2 µL myosin-S1 (10 mg/mL) to the reaction mix, and the decrease in NADH absorbance at 340 nm was recorded for at least 2 min. The reactions were conducted in incremental concentrations of free Ca^2+^. To calculate the amount of ATP hydrolyzed by myosin-S1, an extinction coefficient at 340 nm of 6.22 mM/cm NADH was used.

### 4.5. Protein-Protein Interactions

#### Microscale Thermophoresis (MST)

MST has been applied to study the mutation induced changes in binding affinities between Tn complexes (cTn-WT, cTn-TnC-G34S and cTn-TnI-D127Y) and fluorescently labeled rabbit skeletal actin, or labeled troponin subunits (cTnC-WT, cTnC-G34S, cTnT-WT) and their binding partners. For troponin targeting agents, a binding check was performed to investigate the binding events with fluorescently labeled actin. The labeling procedure was performed according to the manufacturer’s instructions (Monolith NT.115, NanoTemper Technologies). To determine the dissociation constants K_d_, 16 premium treated capillaries (NanoTemper Technologies) were filled with a constant concentration of fluorescently labeled target and an increasing concentration of the ligand. The capillaries were then illuminated by an infrared laser that generates the temperature gradient. The normalized fluorescence (F_norm_ = F_hot_/F_initial_) was plotted against ligand concentration. K_d_ values were estimated by fitting the resultant dose response curves into the K_d_ model (NTanalysis software, NanoTemper).

(1) $$f\left(C\right)=Unbound+\left(Bound-Unbound\right)\;x\frac{C+C_{target}+K_{d}-\sqrt{{\left(C+C_{target}+K_{d}\right)}^{2}-4C\;C_{target}}}{2\;C_{target}}.$$

f(C) is the fraction bound at a given ligand concentration C; Unbound is the F_norm_ signal of the target alone; Bound is the F_norm_ signal of the complex; K_d_ is the dissociation constant or binding affinity; and C_target_ is the final concentration of target in the assay.

### 4.6. Structural Integrity

#### Electron Microscopy

For imaging of reconstituted thin filaments, freshly prepared rabbit skeletal muscle actin was polymerized by addition of 2 mM MgCl_2_, F-actin was then diluted to 0.2 mg/mL followed by reconstitution with cardiac tropomyosin, cardiac troponin complex (cTn-WT, or cTn-G34S, or cTn-D127Y), cMyBPC C0-C2, and myosin-S1 at a molar ratio of 6:1:1:1:1 in the presence of either 20 µM (-)-epigallocatechin-3-gallate (EGCg) or 20 µM levosimendan. Reconstituted thin filaments were then adsorbed to glow-discharged copper grids (400 mesh) with a porous carbon-coat and negatively stained with 1% uranyl acetate. The grids were mounted into a Zeiss transmission electron microscope EM 923 (SESAM), or Zeiss LEO 910. Grids were analyzed at an accelerating voltage of 120 kV, and images were captured and fit using a TemCamF416 device camera with the appropriate software (Tietz Video and Image Processing Systems, Gauting, Germany).

### 4.7. Western Blot Analysis to Evaluate Proteolysis

Homogenized myocardial samples were applied in triplicates on 10% or 12% sodium dodecyl-sulphate polyacrylamide-gels. Proteins were transferred onto PVDF or membrane using a semi-dry technique (TransBlot Turbo™, Bio-Rad, Hercules, CA, USA). The efficiency of protein transfer was checked via UV-induced modification of tryptophane by Tri-chloro-ethanol [65], and subsequent detection of the protein bands on the SDS gel and the appropriate membrane with the GelDoc MP imager (Bio-Rad). Membranes were washed with 1 × TBS containing 0.01% Tween20 (1 × TBST) 3 times for 5 min each and blocked with 3% BSA *w/v* for 1 h at room temperature. Membranes were incubated over night at 4 °C in following primary antibodies.

cMyBPC (1:2000, Invitrogen, PA5-71701), cardiac Troponin I (1:1000, Abcam, Cambridge, UK, 47003), Cathepsin L-33/2 (1:1000, Santa Cruz Biotechnology, Dallas, TX, USA, 32320), Calpain 1 Large Subunit Mu-type (1:1000, Cell Signaling Technology, Danvers, MA, USA, 2556S), Hsp 27 (1:1000, Abcam, 1426), Hsp70 (1:1000, Cell Signaling Technology, 4872), and Alpha B Crystallin (1:1000, Abcam, 76467). Membranes were then washed for 3 × 10 min in 1 × TBST and incubated with the appropriate secondary antibody for 1 h at room temperature. Bands were visualized using the Enhanced Chemiluminescence (ECL) Solution Kit (Bio-Rad) and then imaged in the GelDoc MP (Bio-Rad). Bands were analyzed using ImageJ and Multi Gauge. All signals of proteins were normalized to GAPDH (dilution 1:1000; Sigma) stained on the same blots (Figures S3.4 and S3.5).

### 4.8. Data and Statistical Analysis

Dose response (ATPase-pCa, PM fluorescence intensity-pCa, and IAANS fluorescence intensity-pCa) relations, generated at pCa range from 4.1 to 9.3, were fitted to the Hill equation using SigmaPlot 11.4, Systat Software. A global fit of six to nine measurements was performed. For statistical analysis, Student’s t-test was performed. The differences were considered significant at *p* < 0.05.

## 5. Conclusions/Future Prospects

The patient with the TNNC1 variant p.cTnC-G34S was born already with end stage heart failure and a non-compaction phenotype, indicating that the heart development also might be impaired due to the underlying mutation. Since, in addition, a benign polymorphism in the dystrophin gene (see supplements) was detected, this might have promoted the disease development. Tracking developments in iPS cells would be of great interest for a better understanding. Furthermore, since cTnC is also expressed in slow skeletal muscle, the patient might develop a skeletal muscle disorder at a later stage, which should be taken into consideration. To our knowledge, there is only one study addressing this question. Veltri et al., in 2017, investigated the effects of several HCM variants in cTnC on soleus muscle fibers [66]. Only one of the investigated mutations, namely the cTnC-C84Y mutant, showed impaired Ca^2+^-dependency of isometric force production in soleus muscle fibers. However, more studies including animal models are needed.

The onset of cTnI expression occurs later, with a half-time in infants at around 6 months of age. In the embryonic state, the cardiac isoform of TnI, is not present in the sarcomers; the only TnI form is the skeletal muscle isoform, which is replaced by the cardiac isoform during the first year after birth [28]. In accordance, the patient with the cTnI- D127Y developed RCM at 8 months.

Interestingly, the RCM-TNNI3 variant p.cTnI-D127Y and the end-stage cardiomyopathy TNNC1 p.cTnC-G34S variant showed very similar contractile dysfunctions and effects on thin filament structure and similar responses to levosimendan and EGCg. Both drugs, levosimendan and ECGg, exhibited Ca^2+^ sensitizing and desensitizing effects as expected when using wildtype reconstituted thin filaments. However, these effects were no longer observed with cTnC-G34S and cTnC-D127Y containing thin filaments. Both drugs showed similar beneficial effects concerning thin filament integrity, probably via direct binding to actin and Ca^2+^ sensitivity of thin filament activation, which were clearly more prominent for EGCg. However, more detailed investigations using cell culture or animal models are needed.

So, why did different phenotypes develop despite the similar effects of both variants? This is a general problem for all sarcomeric gene mutations and cardiomyopathies [2,12]. Besides the different expression time points of the troponin subunits during development and early childhood, post-translational modifications [12], epigenetics [67], and the presence of troponin subunits in the nuclei linked to differentiation and regeneration [68,69] could contribute specifically to the development of a particular phenotype. Additional factors, such as the distribution of the mutant isoforms within the heart due to burst-like expression [70] and differing expression levels of the mono-allelic mutants versus the corresponding wild type proteins, contribute to the development of specific phenotypes. Hence, which factors are involved and to which extent remains largely unknown and is an important subject for future investigation.

Limitation of the study: The patient and the donor group show a lot of variations in the western blot, although the same amount of the protein from each sample has been loaded. This could be due to the variation between the individuals based on the disease stage, age, gender, the medications, and/or the cause of death of each individual from the donor group. However, as we have normalized the western blots to GAPDH, we believe this comparison is valid and accurate.

## Figures and Tables

**Table 1:** Summary of pCa_50_ values and nHill slopes measured by Ca^2+^-dependent force measurements of skinned fibers or skinned cardiomyocytes.

	pCa_50_	nHill	Min	Max
G34S Cardiomyocytes	6.373 ± 0.096 ***	1.378 ± 0.360	4.21 ± 1.01	44.98 ± 2.00 ᴥᴥᴥ
Donor Cardiomyocytes	5.911 ± 0.051 ***	1.046 ± 0.147	2.67 ± 0.12	64.24 ± 2.23 ᴥᴥᴥ
D127Y Skinned fibers	6.027 ± 0.021 ⸸⸸⸸	2.020 ± 0.179	7.383 ± 0.679	10.17 ± 2.61
WT Skinned fibers	5.633 ± 0.013 ⸸⸸⸸	3.198 ± 0.264	6.337 ± 0.243	15.38 ± 2.97

⸸⸸⸸ *p*-value = <0.001; *** *p*-value = <0.001; ᴥᴥᴥ *p*-value = <0.001.

**Table 2:** Summary of y_min_, y_max_, and Amp values of the actomyosin-S1-ATPase activity. y_min_ and y_max_ designate the basal (at low Ca^2+^) and the maximal ATPase activity (at hight Ca^2+^), respectively. Amp designates the amplitude, i.e., the difference between minimal and maximal ATPase activity, measured with reconstituted thin filaments (TF) in the presence of the N-terminal fragment of Myosin binding protein C (cMyPBC) alone or with the troponin targeting drugs levosimendan or EGCg.

		TF + cMyBPC	TF + cMyBPC + Levosimendan	TF + cMyBPC + EGCg
WT	*y_min_* *Amp*	0.074 ± 0.025 0.381 ± 0.042	0.107 ± 0.035 0.497 ± 0.06	0.065 ± 0.038 0.547 ± 0.021 ‡
	*y_max_*	0.455 ± 0.017 *⸹	0.604 ± 0.025 *	0.612 ± 0.024 ⸹
G34S	*y_min_* *Amp*	0.107 ± 0.025 0.41 ± 0.041	0.120 ± 0.022 0.369 ± 0.037	0.114 ± 0.037 0.489 ± 0.061
	*y_max_*	0.517 ± 0.016 ᴥ	0.489 ± 0.015	0.603 ± 0.024 ᴥ
D127Y	*y_min_* *Amp*	0.110 ± 0.064 0.427 ± 0.095	0.107 ± 0.031 0.419 ± 0.055	0.124 ± 0.031 0.388 ± 0.05 ‡
	*y_max_*	0.537 ± 0.031 ⸸	0.526 ± 0.024 ⸸	0.512 ± 0.019

‡ Significant difference, *p*-value = 0.022; * Significant difference, *p*-value = 0.0005; ⸸ Significant difference, *p*-value = 0.048; ⸹ Significant difference, *p*-value = 0.0003; ᴥ Significant difference, *p*-value = 0.020.

**Table 3:** Summary of pCa_50_ values and nHill slopes measured by Tm-PM fluorescence in the presence and absence of troponin targeting agents.

		TF + cMyBPC	TF + S1 + cMyBPC	TF + S1 + cMyBPC + Levosimendan	TF + S1 + cMyBPC + EGCg
WT	pCa_50_	7.32 ± 0.16	7.49 ± 0.10	7.55 ± 0.09	7.16 ± 0.05 §
	nHill	1.00 ± 0.28	0.95 ± 0.17	1.16 ± 0.21	2.07 ± 0.66
G34S	pCa_50_	7.06 ± 0.06	7.12 ± 0.08 *	7.35 ± 0.06 †	7.48 ± 0.07 ‡ᴥ
	nHill	1.66 ± 0.43	1.29 ± 0.27	1.27 ± 0.17 ††	1.69 ± 0.27
D127Y	pCa_50_	7.03 ± 0.06	7.04 ± 0.06 ⸸	7.44 ± 0.08	7.34 ± 0.08 ⸹
	nHill	1.44 ± 0.28	1.22 ± 0.19	1.52 ± 0.26	1.53 ± 0.30

For statistical analysis, Student’s t-test was performed, *n* = 5–9; * Significant difference vs. TF WT + S1 + cMyBPC, *p* = 0.021; ⸸ Significant difference vs. TF WT + S1 + cMyBPC, *p* = 0.004; † Significant difference vs. TF G34S + S1 + cMyBPC, *p* = 0.040; †† Significant difference vs. TF D127Y + S1 + cMyBPC, *p* = 0.003; § Significant difference vs. TF WT + S1 + cMyBPC, *p* = 0.011; ‡ Significant difference vs. TF G34S + S1 + cMyBPC, *p* = 0.010; ᴥ Significant difference vs. TF WT + S1 + cMyBPC + EGCg, *p* = 0.005; ⸹ Significant difference vs. TF D127Y + S1 + cMyBPC, *p* = 0.017.

**Table 4:** Statistical analysis of y_max_ values of thin filaments measured by NADH coupled ATPase assay in the presence and absence of levosimendan or EGCg compared to their respective wild type filaments.

	*y_max_* TF + cMyBPC + Levosimendan	*p*-Value vs TF WT + cMyBPC + Levosimendan	*y_max_* TF + cMyBPC + EGCg	*p*-Value vs TF WT+ cMyBPC + EGCg
G34S	0.489 ± 0.015 **	0.002	0.603 ± 0.024	0.798
D127Y	0.526 ± 0.024 *	0.048	0.512 ± 0.019 **	0.009

* *p* < 0.05/** *p* < 0.01, *n* = 6–9.

**Table 5:** Summary of clinical, functional, and structural characterization differences in cTnC G34S and cTnI D127Y variants.

Parameter	cTnC-G34S	cTnI-D127Y
Phenotype	NCM	RCM
Age of onset/diagnosis	congenital	8 months
Outcome	transplanted	deceased
Histology	Mild fibrosis, myofbrillar loss, locally red. cTnC expression	Fibrosis, structural disturbances between cardiomyocytes
Ca^2+^-sensitivity of cardiomyocytes/myofibers	Increased, max. tension reduced	Increased max. tension preserved
Ca^2+^-sensitivity of reconstituted filaments (Myosin S1 ATPase and Tm-PM fluor.)	No increase	No increase
Protein quality control	Increased Hsp27, αβ-crystallin and cathepsin, reduced Hsp70	No data available
Interactions within Tn complex	cTnC-cTnT affinity decreased	cTnI-cTnC and cTnI-cTnT affinity increased
Interaction with actin and incorporation in thin filaments	normal	normal
Thin filament structure	Fragmented, short, bundled, S1 decoration reduced	Fragmented, short, bundled, S1 decoration reduced
Effects of levosimendan and EGCg on Ca^2+^-dependent activation	Decreased max. S1 ATPase activity with levosimendan, but increase with EGCg, increased Ca^2+^-sensitivity (Tm-PM assay)	Decreased max. S1 ATPase activity with levosimendan and EGCg, increased Ca^2+^-sensitivity (Tm-PM assay)
Effects of levosimendan and EGCg on thin filament structure	Less fragmentation, longer filaments, but increased bundling. Restored decoration with myosin S1 with EGCg	Less fragmentation, longer filaments, but increased bundling. Restored decoration with myosin S1 with EGCg

## Data Availability

Data is contained within the article.

## Supplementary Materials

The following are available online at https://www.mdpi.com/article/10.3390/ijms22179625/s1.
