# Supplementary material for: De Novo Missense Mutations in TNNC1 and TNNI3 Causing Severe Infantile Cardiomyopathy Affect Myofilament Structure and Function and Are Modulated by Troponin Targeting Agents
Source: Int J Mol Sci. 2021 Sep 6;22(17):9625. doi: 10.3390/ijms22179625 (PMC8431798; doi:10.3390/ijms22179625)
Supplement: Supplementary file 1 [file ijms-22-09625-s001.zip › ijms-1347646-supplementary.pdf]

De *novo* missense mutations in *TNNC1* and *TNNI3* causing severe infantile cardiomyopathy affect myofilament structure and function and are modulated by troponin targeting agents

Roua Hassoun[1,2], Heidi Budde[1,2], Hans Georg Mannherz[1,3], Mária Lódi[4], Setsuko Fujita-Becker[5], Kai Thorsten Laser[6], Anna Gärtner[7], Karin Klingel[8], Desirée Möhner[9], Robert Stehle[9], Innas Sultana[1,2], Thomas Schaaf[1,2], Mario Majchrzak[1,2], Verena Krause[1,2], Christian Herrmann[10], Marc M. Nowaczyk[11], Andreas Mügge[1,2], Gabriele Pfitzer[9], Rasmus. R. Schröder[5], Nazha Hamdani[1,2], Hendrik Milting[7], Kornelia Jaquet[1,2,*], Diana Cimiotti[1,2,12,*]

1 Institut für Forschung und Lehre (IFL), Molecular and Experimental Cardiology, Ruhr University Bochum, 44801 Bochum, Germany

2 Department of Cardiology, St. Josef-Hospital and Bergmannsheil, University Clinic of the Ruhr University Bochum, 44801 Bochum, Germany

3 Department of Anatomy and Molecular Embryology, Ruhr University Bochum, 44801 Bochum, Germany

4 Department of Neuroanatomy and Molecular Brain Research, Ruhr University Bochum, Medical Faculty, 44801 Bochum, Germany

5 Cryoelectron Microscopy, Bioquant, Medical Faculty, University of Heidelberg, 69120 Heidelberg, Germany

6 Center for Congenital Heart Disease/Pediatric Cardiology Heart and Diabetes Centre NRW, University Clinic of the Ruhr University Bochum, 32545 Bad Oeynhausen, Germany

7 Erich and Hanna Klessmann Institute, Heart and Diabetes Centre NRW, University Hospital of the Ruhr University Bochum, 32545 Bad Oeynhausen, Germany

8 Institute for Pathology and Neuropathology, University Hospital Tuebingen, 72076 Tuebingen, Germany

9 Institute of Vegetative Physiology, University of Cologne, 50931 Cologne, Germany

10 Department of Physical Chemistry I, Ruhr University Bochum, 44801 Bochum, Germany

11 Plant Biochemistry, Faculty of Biology and Biotechnology, Ruhr University Bochum, 44801 Bochum, Germany

12 Department of Clinical Pharmacology, Ruhr University of Bochum, 44801 Bochum, Germany

Correspondence: Kornelia Jaquet: kornelia.jaquet@rub.de, Tel: +49-234-3227639 Diana Cimiotti: Diana.Cimiotti@rub.de, Tel: +49-234-3227639

* both authors equally contributed to this work

Supplementary tables

1. Genetical analysis

Table S1.1 Genetic analysis and variant classification of p.cTnC-G34S patient.

n.a. designates not available, dbSNP ID the database identity number for single nucleotide polymorphism, ACMG (American College of Medical Genetics and Genomic) based classification 4 means likely pathogenic and 3 of unknown significance.

Gene	RefSeq ID mRNA	RefSeq ID Protein	dbSNP ID	ACMG
TNNC1	NM_003280.2:c.100G>A	NP_003271.1:p.Gly34Ser	n.a.	4
TTN	NM_001267550.1:c.104522G>A	NP_001254479.1:p.Arg34841His	rs373709706	3
TTN	NM_001267550.1:c.68525T>C	NP_001254479.1:p.Ile22842Thr	rs368301580	3
DMD	NM_004006.2:c.8147A>G	NP_003997.1:p.Gln2716Arg	rs750640802	3

Table S1.2 Genetic analysis and variant classification of p.cTnI-D127Y patient.

n.a. designates not available, dbSNP ID the database identity number for single nucleotide polymorphism, ACMG (American College of Medical Genetics and Genomic) based classification 4 means likely pathogenic and 3 of unknown significance

Gene	RefSeq ID mRNA	RefSeq ID Protein	dbSNP ID	ACMG
TNNI3	NM_000363.4:c.379G>T	NP_000354.4:p.Asp127Tyr	n.a.	4
ANK2	NM_001148.4:c.8626G>T	NP_001139.3:p.Val2876Phe	n.a.	3

2. Function

Table S2.1. Summary of pCa_{50} values and nHill slopes measured by ATPase coupled assay.

Errors are given as mean standard errors (±SEM). n = 4-7

		TF+cMyBPC	TF+cMyBPC+Levosimendan	TF+cMyBPC+EGCg
**WT**	pCa_{50}	7.041±0.075	6.889±0.101	6.911±0.088
	nHill	2.243±0.947	1.323±0.374	1.742±0.527
**G34S**	pCa_{50}	7.116±0.06	7.053±0.063	7.114±0.083
	nHill	2.505±1.263	2.350±0.983	2.868±2.486
**D127Y**	pCa_{50}	7.192±0.220	7.023±0.107	7.059±0.071
	nHill	1.452±0.884	1.441±0.494	3.607±2.779

Table S2.2 K_d values obtained from MST measurements of troponin subunits.

Labeled cTnC 120 [nM]	cTnT	cTnI	$K_d \pm K_d$ Confidence
cTnC WT	—	cTnI WT[*] 66.7 to 0.00203 [μM]	179.57 ± 47.22 [nM]
cTnC WT	—	cTnI D127Y[*] 62.5 to 0.00191 [μM]	1.32 ± 0.41 [nM]
cTnC WT	cTnT WT[ᴥ] 58.3 to 0.00178 [μM]	—	2.92 ±1.21 [μM]
cTnC G34S	—	cTnI WT 41.7 to 0.00222 [μM]	186.43 ± 15.09 [nM]
cTnC G34S	cTnT WT[ᴥ] 73.6 to 0.00449 [μM]	—	14.28 ± 6.62 [μM]
—	Labeled cTnT WT 60 [nM]	cTnI WT[†] 10 to 0.00061 [μM]	109.67 ± 24.45 [nM]
—	Labeled cTnT WT 60 [nM]	cTnI D127Y[†] 5 to 0.000305 [μM]	43.80 ± 14.81 [nM]

* Significant difference, *p-value = 0.0128, n* = 3-9
† Significant difference, *p-value = 0.0363, n* = 3-9
ᴥ Significant difference, *p-value = 0.001, n* = 3-9

Table S2.3. K_d values obtained from MST measurements of troponin complexes and actin.

	cTn 53.5 to 0.00157 [μM]	$K_d \pm K_d$ Confidence [μM]
Labeled Actin 20 [nM]	cTn WT	2.01 ± 0.70
Labeled Actin 20 [nM]	cTn G34S	1.93 ± 0.96
Labeled Actin 20 [nM]	cTn D127Y	2.20 ± 0.68

n = 3-9

Supplementary figures

1. Clinics and genetics

Figure S1.1 Clinical characterization of the patients by Echocardiography and X-Ray findings

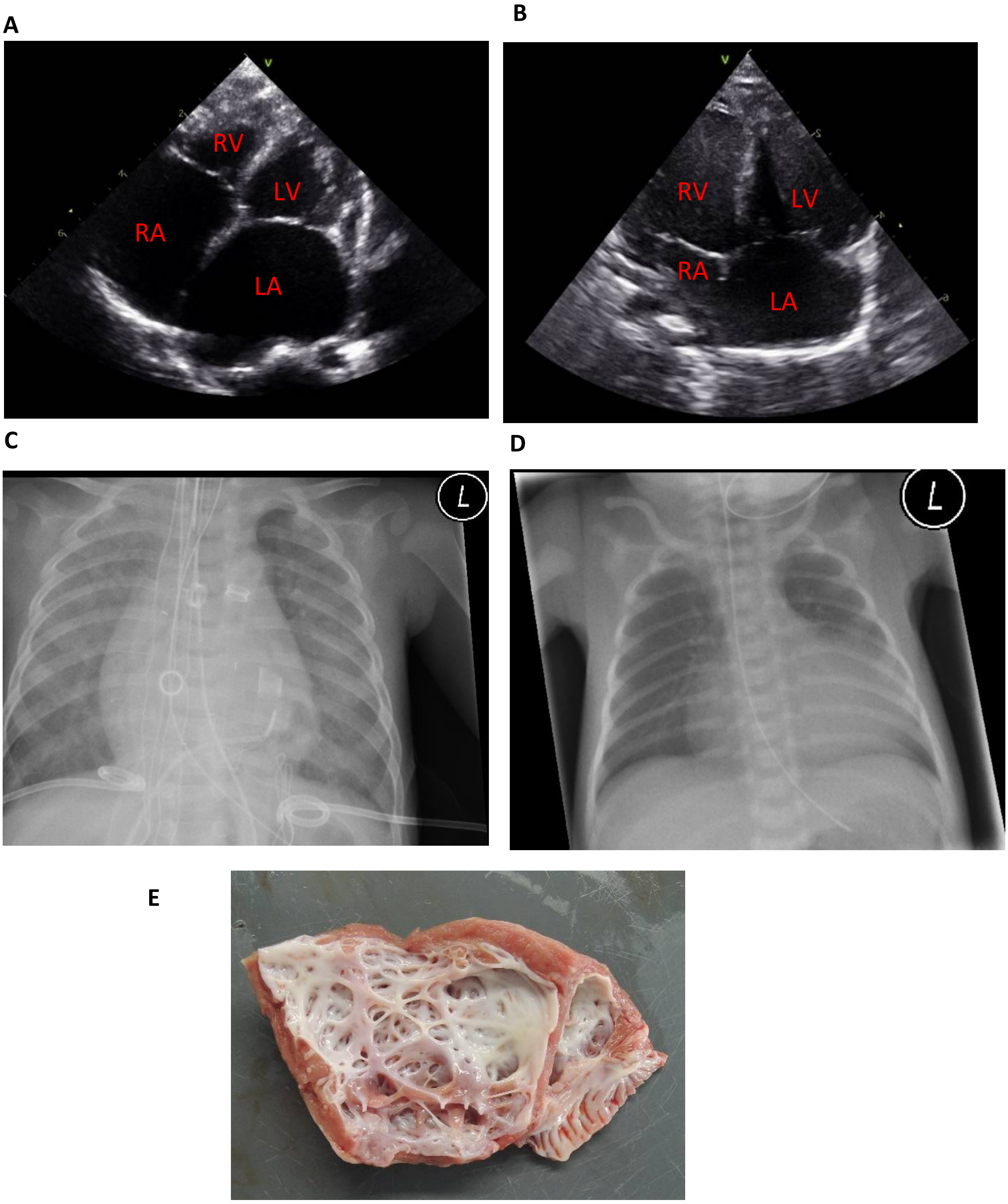

Figure S1.1. **A.** Patient cTnI-D127Y: Apical B-mode four chamber view using standard echocardiography. RA=right atrium, RV=right ventricle, LA=left atrium, LV=left ventricle. Both atria are enlarged, the ventricles remain in normal size with restrictive physiology. **B.** Patient cTnC-G34S: Apical B-mode four chamber view using standard echocardiography. RA=right atrium, RV=right ventricle, LA=left atrium, LV=left ventricle. **C.** Patient cTnI-D127Y: Cardiomegaly with cardiothoracic ratio of 0,63. Absent cardiac waist, expanded bronchial bifurcation, mild global decrease in transparency as a sign for pulmonary congestion. **D.** Patient cTnC-G34S: Cardiomegaly with left ventricular enlargement predominating the left structural edge, cardiothoracic ratio of 0,67, mild signs of pulmonary congestion. **E.** ventricular cross section of the cTnC G34S patients transplanted heart.

Figure S1.2. Sequencing chromatograms of p.cTnC-G34S and p.cTnI-D127Y patients' DNA.

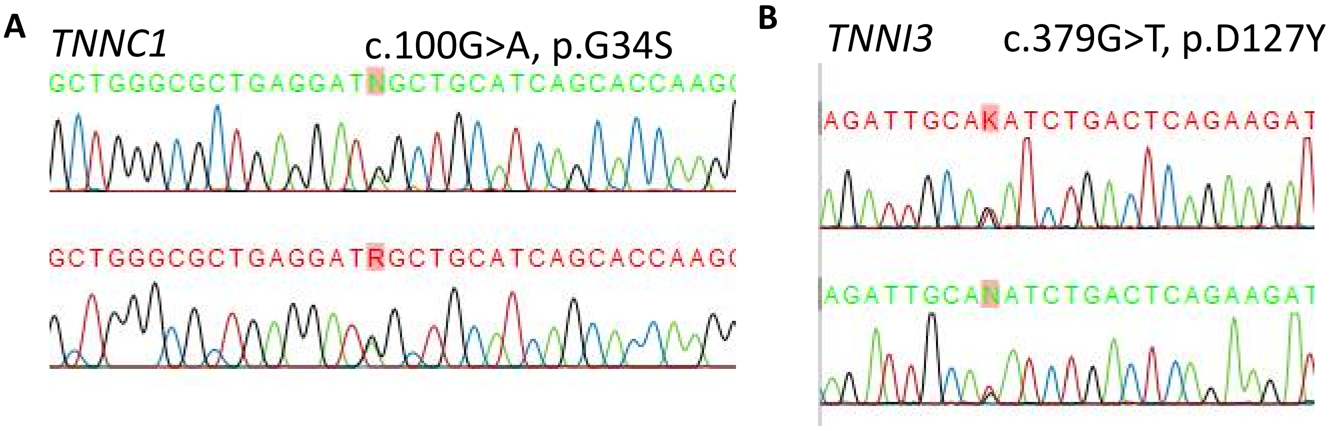

Figure S1.3 Genetic screening of the patients' family members

A

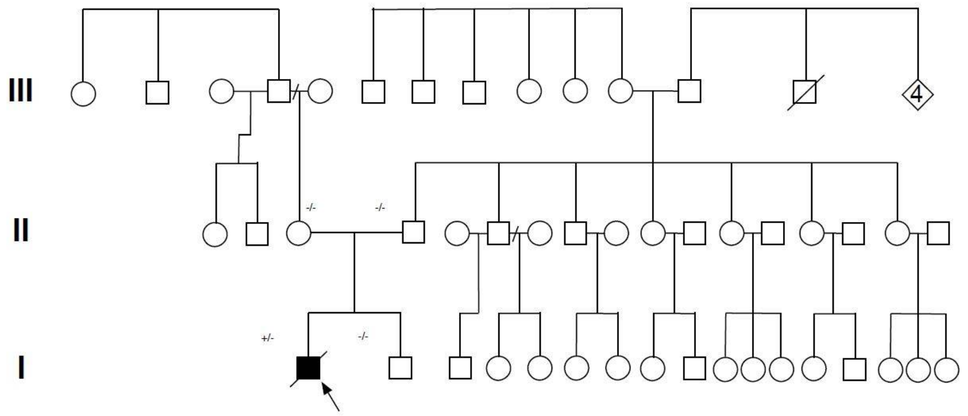

B

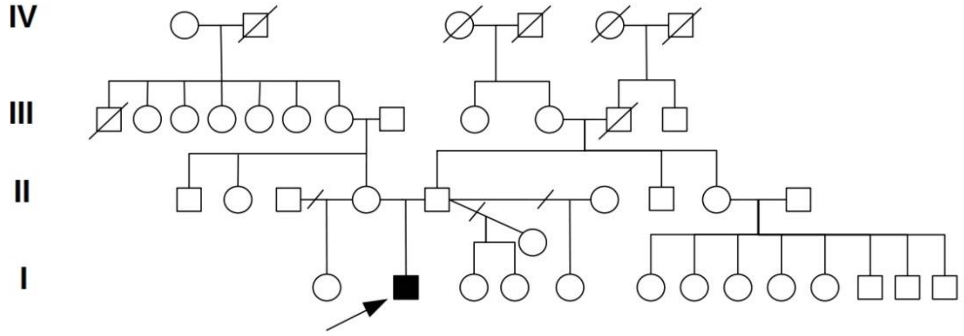

Figure S1.3. Pedigrees of **A.** cTnI-D127Y and **B.** cTnC-G34S patients' families. Squares indicate males, circles: females, open symbols indicate unaffected subjects, solid symbols: affected individuals, slanted bars: deceased individuals.

Figure S1.4. Clinical characterization of the patients using histology

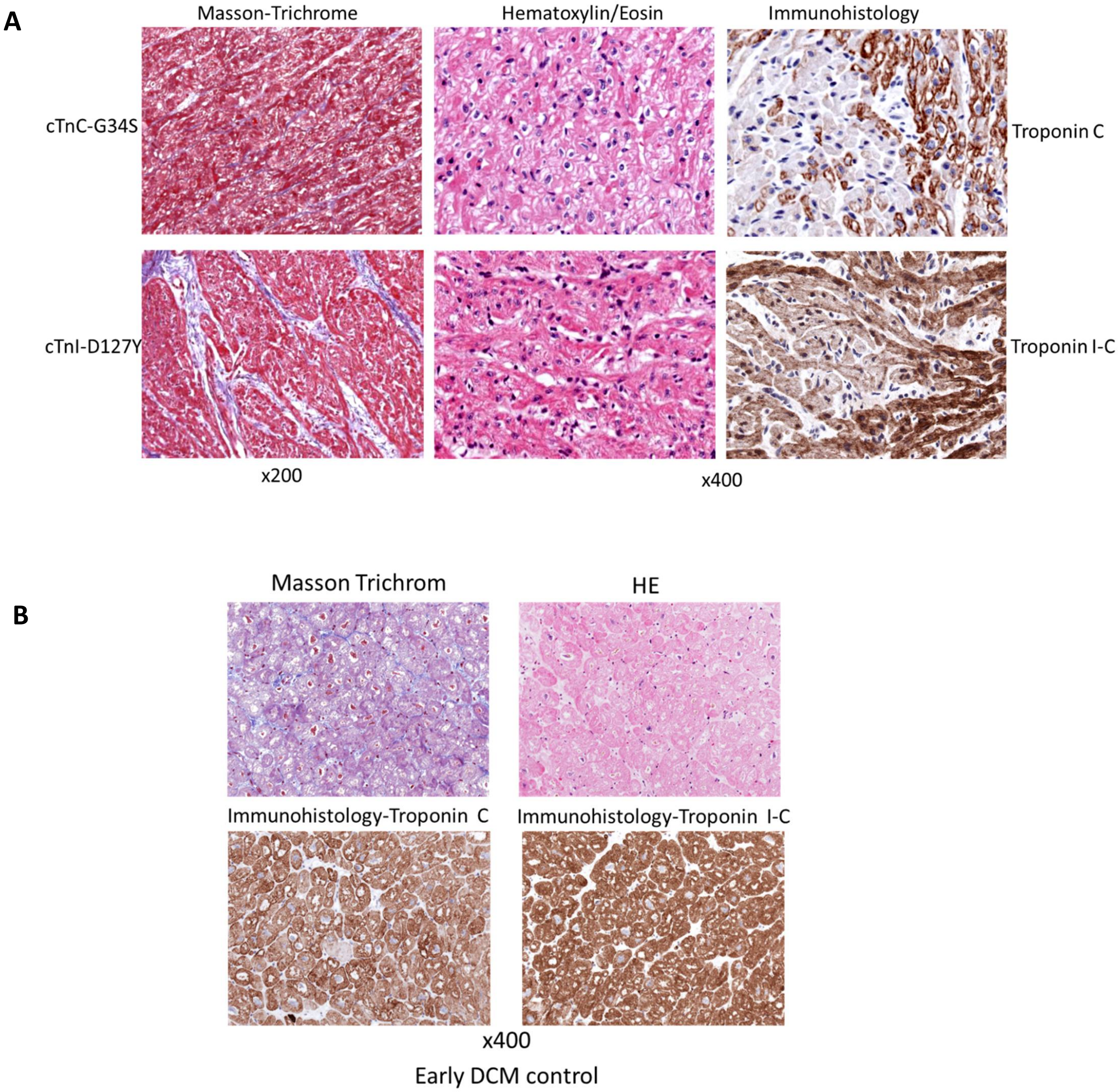

Figure S1.4. Histology of biopsy samples from the hearts of the cTnC-G34S and cTnI D127Y patients (A) and early stage of DCM control (B). Different staining as Masson Trichrom (muscle tissue red, collagen. blueish) and H/E (hematoxylin/eosin; cytoplams red, nuclei dark violet) were applied. In addition, immunhistochemistry was performed using anti - cardiac troponin C and anti-cardiac troponin I antibodies.

2. Protein isolation and purification

Figure S2.1. Isolation of skeletal actin, cardiac tropomyosin, cardiac myosin-S1, and cMyBPC C0-C2

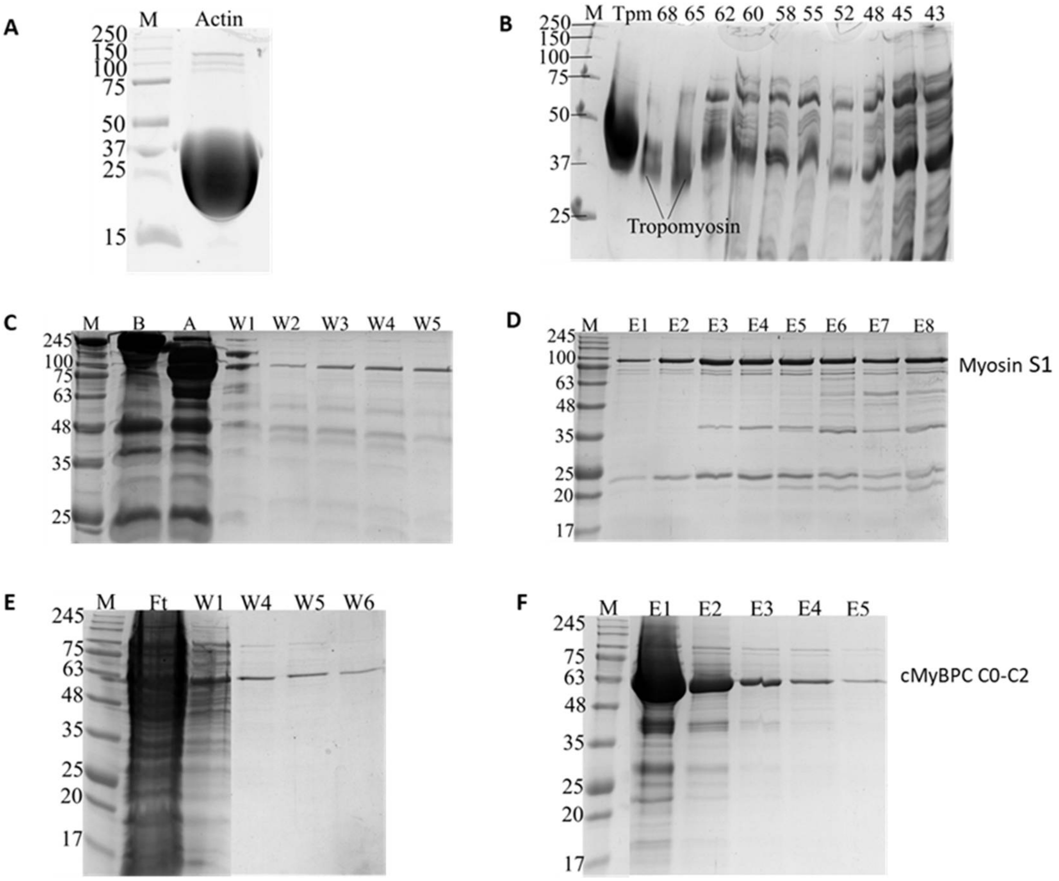

Figure S2.1. Representative 12% SDS gel images of **A**: Actin isolation, **B:** Purification of Tropomyosin, Tpm: Tropomyosin standard; Eluted fractions:43-68. **C**: myosin-S1 purification using DEAE cellulose: second lane: full length myosin before digestion; third lane: after digestion and before DEAE purification; W1-W5: Washing samples; **D**: E1-E8: Eluted fractions, **E**: Isolation of His tagged MyBP-C C0-C2, Ft: Flowthrough; W1-W6: Washing samples, **F**: E1-E5: Eluted fractions. M is the protein standard (band sizes are given in kDa).

Figure S2.2. cTnI-D127Y, cTnC-G34S, cTnT-WT isolation, and reconstitution of troponin complex

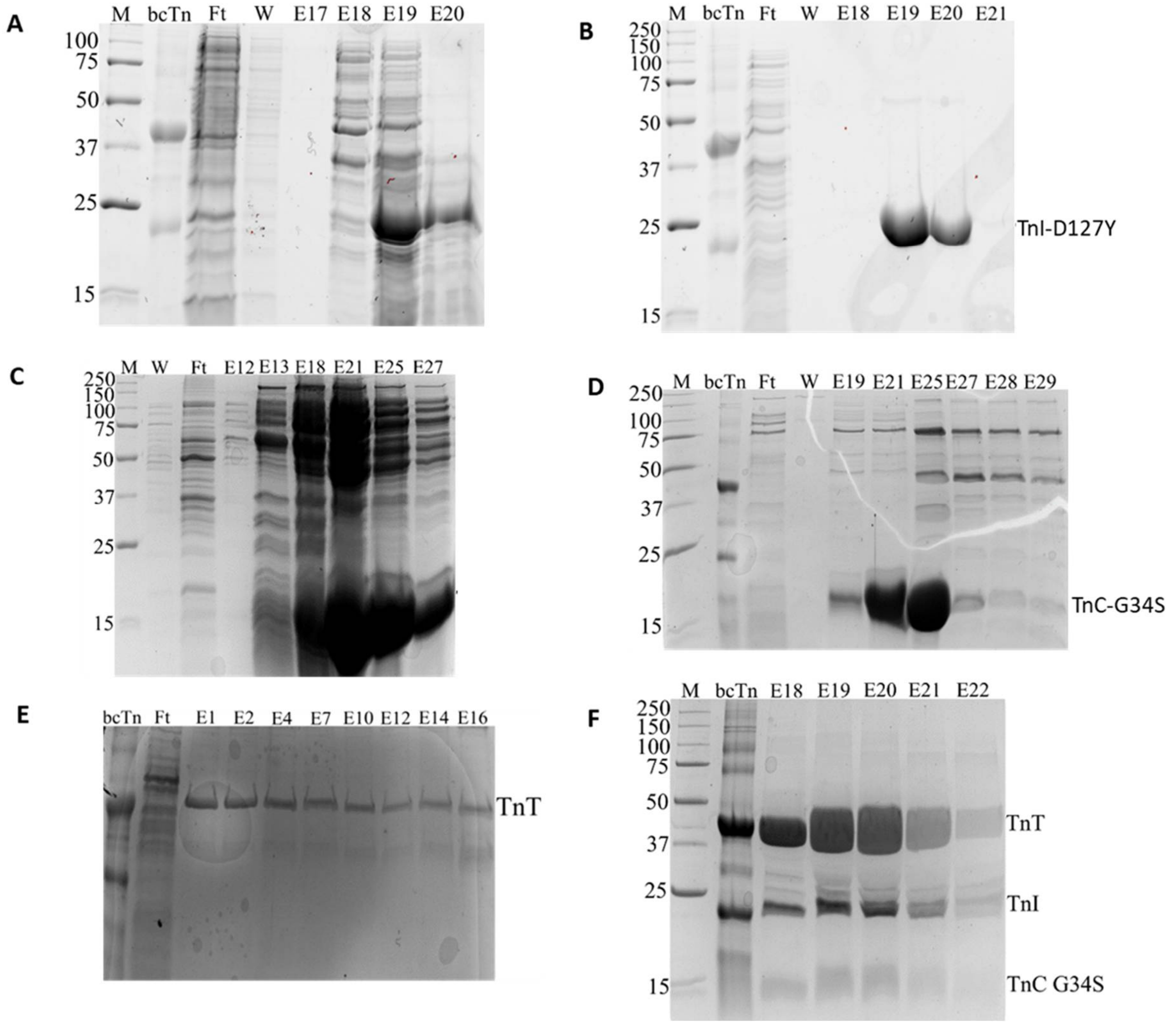

Figure S2.2. Representative 12% SDS gel images of **A:** cTnI-D127Y purification using CM Sepharose; W: washing sample; E17-E20: eluted fractions. **B:** cTnI-D127Y purification using TnC-affinity column, W: washing sample; E18-E21: eluted fractions; Ft: flow through, **C**: cTnC-G34S purification; W: washing sample; Ft: flow through; E12-E27: eluted fractions from DE 52, **D**: E19-E29: eluted fractions from Phenylsepharose 4B. **E:** cTnT-wt purification by DE52 cellulose; E1-E16: eluted fractions. **F.** Gel filtration of troponin complex cTn-G34S; E18-E22: eluted fractions; bcTn: bovine cardiac troponin; M is the protein standard (band sizes are given in kDa).

Functional analysis, interactions

Figure S3.1 IAANS fluorescence measurements of thin filaments containing wild type or cTnC-G34S

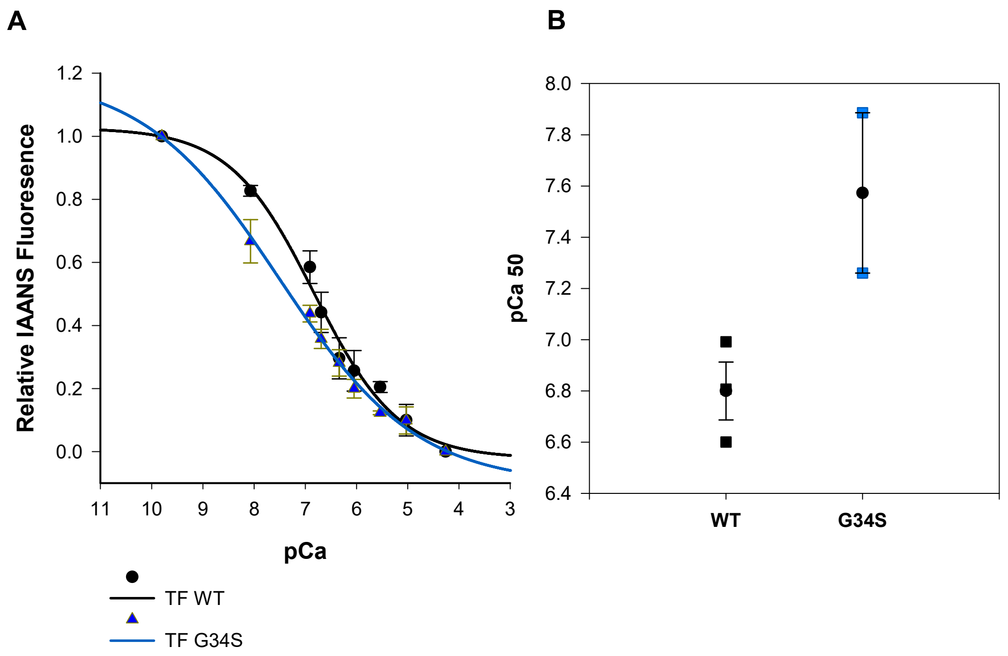

Figure S3.1. A. Representative data for the Ca^{+2}-dependent decrease in fluorescence intensity of thin filaments reconstituted with (cTn-TnC WT- IAANS) versus (cTn-TnC G34S-IAANS), data are given as normalized fluorescence ±SEM (*n*=3-4) vs. the negative logarithm of the Ca^{2+} concentration (pCa), fitted to the Hill equation. **B.** Ca^{2+} sensitivity, TF-WT pCa_{50}=6.82 ± 0.14, TF-G34S pCa_{50}=7.49 ± 0.27, * indicates statistical significance with $p<0.05$.

Figure S3.2 Degree of replacement of endogenous (murine) by the recombinant (human) Tn-complex in fibres.

A

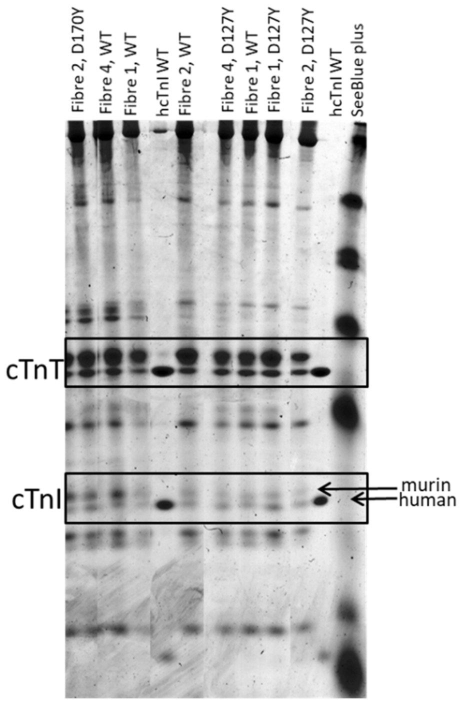

Complex	Exchange efficiency (%)
D127Y	28
WT	24
WT	26
WT	45
D127Y	55
WT	50
D127Y	57
D127Y	56

B

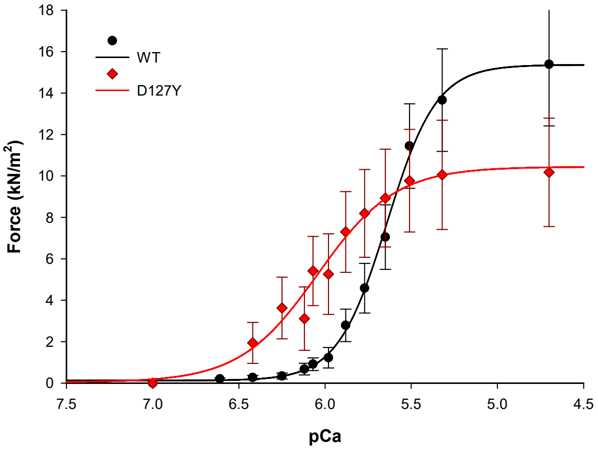

Figure S3.2 A. After force measurements, fibres were fixed with 15% (w/v) TCA, homogenized, and loaded on a prolonged SDS gel (12.5% AA). The extended run distance in combination with a longer and slower running time (5 mA, 45 V, 18 h) led to a greater resolution of proteins in the range of 25-40 kDa. Gel was stained with Coomassie Brilliant Blue R-250 and the relative amounts of the endogenous and recombinant TnI-band were determined by densitometry. B. Force-pCa relation of guinea pig skinned fibres after exchanging endogenous troponin with human cardiac troponin containing either wild type cTnI or TnI-D127Y. Data are presented as mean ±SEM, *n*=6 for cTnI-WT and *n*=4 for cTnI-D127Y vs pCa (negative logarithm of the free Ca^{2+}-concentration).

Figure S3.3. Co-sedimentation assay (A) using Tn-WT and Tn-G34S in presence and absence of Ca^{2+} (B) using Tn-D127Y

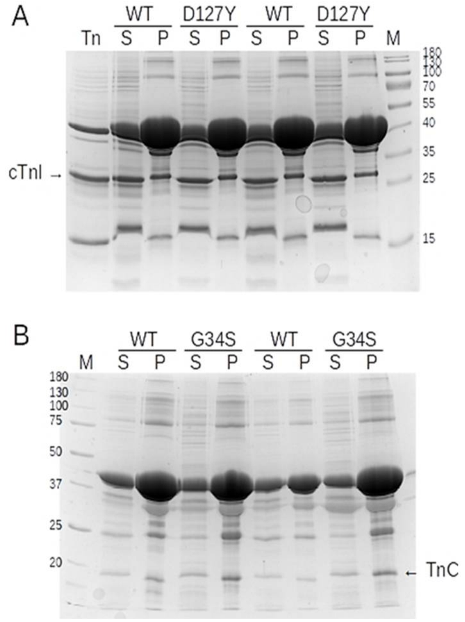

Figure S3.3: Representative Coomassie stained gels of the cosedimentation of cTn containing cTnI-D127Y (A) or cTnC-G34S (B). M: protein marker (band sizes are given in kDa), WT: cTn wildtype, D127Y: cTn containing cTnI-D127Y, G34S: cTn containing cTnC-G34S, S: supernatant, P: pellet. Tn: bovine cardiac troponin standard.

Figure S3.4. Western blot analysis of HSPs

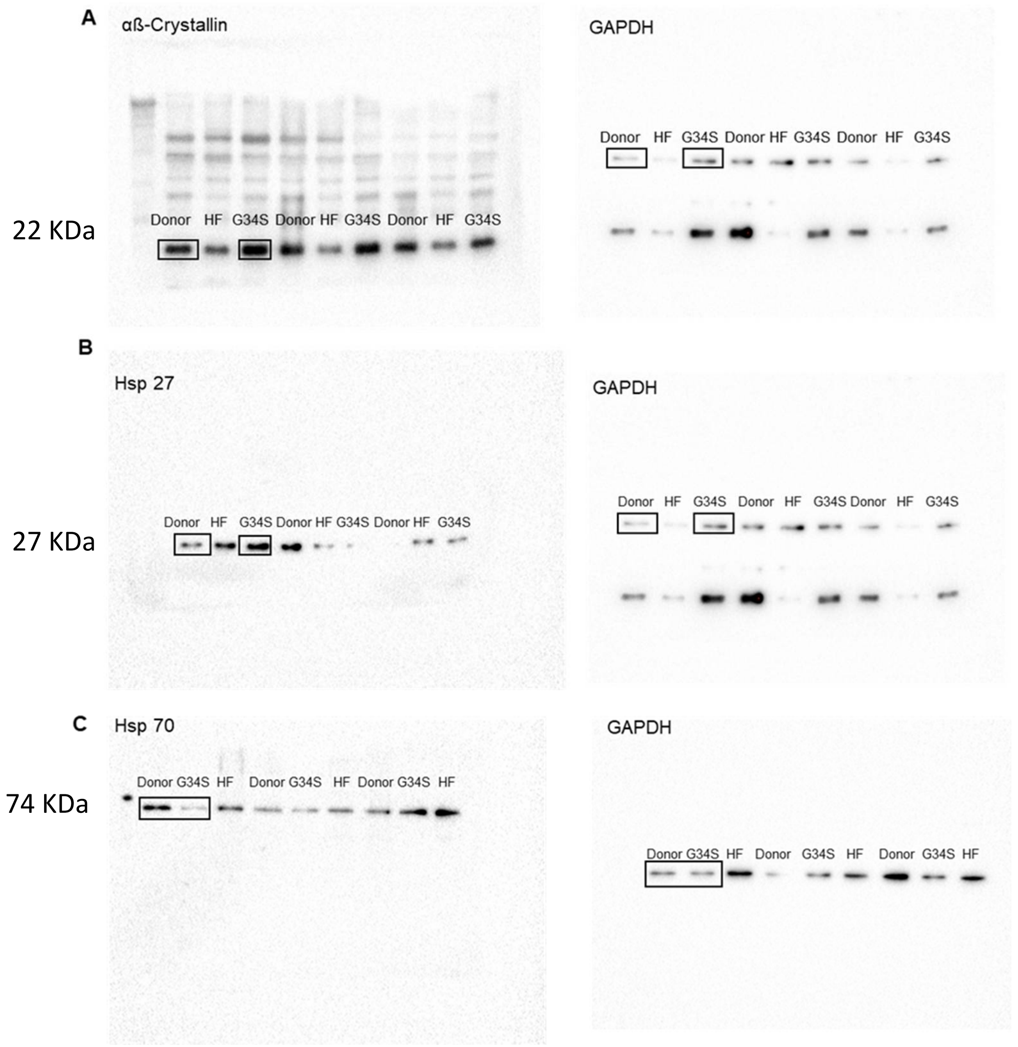

Figure S3.4. A. αß-crystallin protein levels in donor and TnC-G34S tissue samples **B.** Hsp 27 protein levels in donor and TnC-G34S tissue samples. **D.** Hsp 70 protein levels in donor and TnC-G34S tissue samples. Data are shown as mean±SEM; *n*=5 samples/group.

Figure S3.5. Western blot analysis of proteolysis, cTnI and cMyBPC protein levels

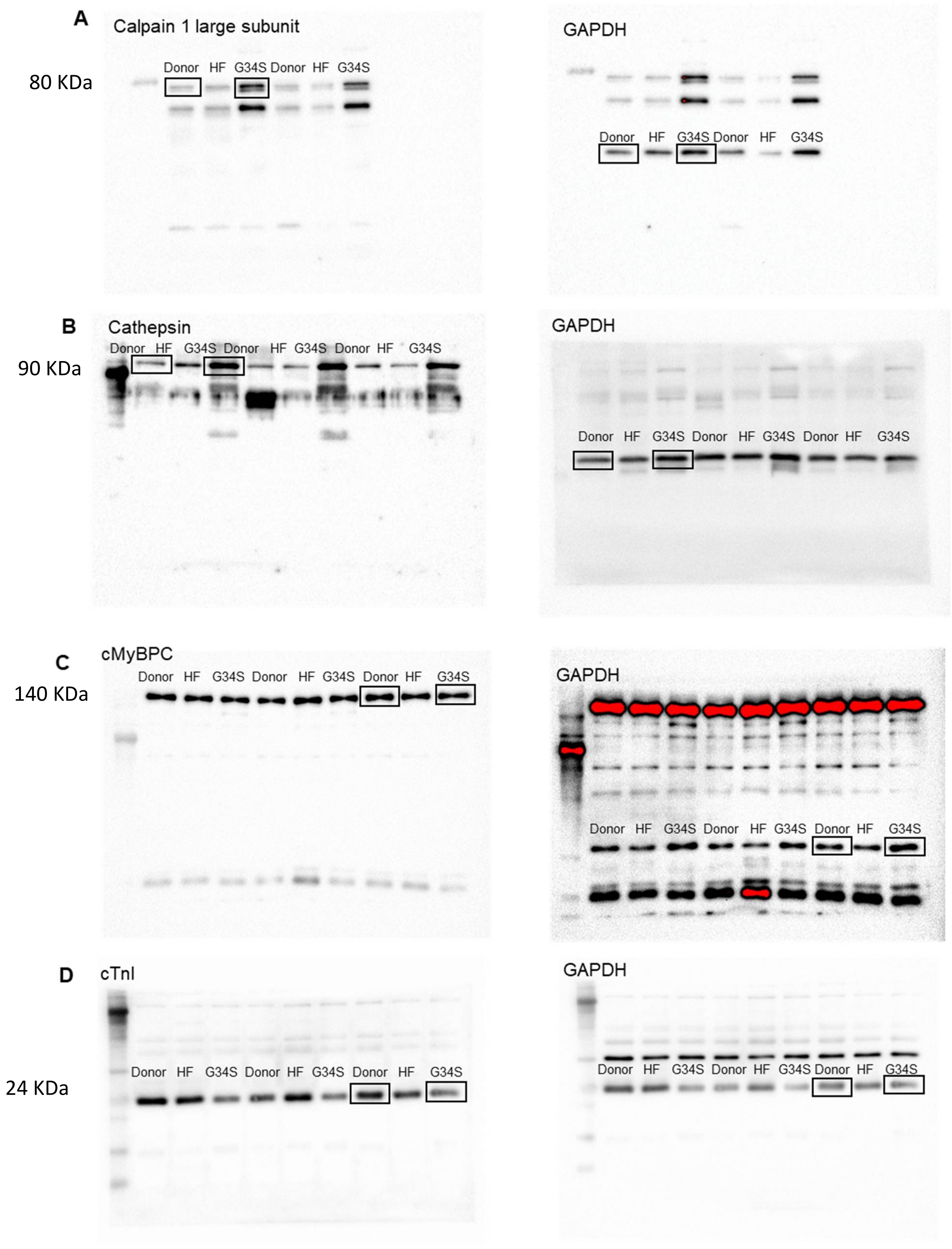

Figure S3.5. A. Calpain 1 protein levels. **B.** Cathepsine protein levels. **C.** cMyBPC protein levels **D.** cTnI protein levels. Data are shown as mean±SEM; *n*=5 samples/group.

Supplementary methods

Histology and Immunohistology

Myocardial tissue was fixed in 4% phosphate-buffered formaldehyde and embedded in paraffin. Four um thick tissue sections were stained with Masson-Trichrome and hematoxylin-eosin and examined by light microscopy. Troponin C was stained by using a mouse monoclonal antibody against troponin C (Clone 7B9, 1:400), and for the detection of troponin I, a monoclonal mouse anti-human Troponin I-C (Clone C5, 1:400), both Santa Cruz Biotechnology, Heidelberg, Germany, was used. Immunohistochemical analysis was performed on an automated immunostainer following the manufacturer's protocol (Benchmark; Ventana Medical Systems, Tucson, AZ) and using the ultraView detection system (Ventana) and diaminobenzidine as substrate. Tissue sections were counterstained with hematoxylin.

IAANS fluorescence measurements of thin filaments containing wild type or cTnC-G34S

To investigate the effects of cTnC G34S mutation on Ca^{+2} binding at the regulatory site II in vitro, the change in fluorescent intensity of a 2-[4-(iodoacetamido) aniline] naphthalene-6-sulfonate (IAANS) label bound to cTnC subunits (in both the wild type and mutant cTnC) was monitored.

The fluorescence of IAANS-labeled proteins dependent on the Ca^{2+} concentration was measured at 15 °C using a PerkinElmer LS 55 spectrofluorometer. IAANS was excited at 330 nm and emission was monitored at 450 nm. Microliter amounts of $CaCl_2$ solution in the concentrations of (10 mM, 4 mM and 2 mM) were titrated to 200 µl assay mix volume of (1,05µM Actin, 0.15µM tropomyosin, 0.15µM troponin complex, 1mM ATP) in a titration buffer containing (200 mM MOPS (pH 7.5), 150 mM KCl, 3 mM $MgCl_2$, 1 mM DTT, 0.02% Tween-20, and 2 mM EGTA). The fluorescence intensity was tested at different free [Ca^{+2}] (pCa 10, pCa 8.07, pCa 6.91, pCa 6.69, pCa 6.34, pCa 6.05, pCa 5.54, pCa 5.03, pCa 4.26). The relative fluorescence intensity was plotted as a function of pCa concentration (Excel, Microsoft). The resultant curves were fitted to the Hill equation (Dynamic non-linear regres-sion) using SigmaPlot 11.2 software. (a global fit for the mean of three to five measurements ± SE was performed).

$$Ynorm = Ymax + \frac{\min - max}{(1 + 10^{n\cdot(pCa - pCa50)})}$$

Y norm: The normalized fluorescence intensity; *Y min:* The minimum fluorescence intensity, *Y max:* The maximum fluorescence intensity, *pCa_{50}:* The pCa at half-maximum fluorescence intensity; *n:* Hill Coefficient.

Cosedimentation

G-actin was polymerised by addition of 100 mM KCl, 1 mM $MgCl_2$ and 1 mM ATP. Thin filaments were reconstituted in filament buffer (20 mM HEPES, pH 7.5; 70 mM KCl; 5 mM $MgCl_2$, 500 µM EGTA; 2 mM DTT) from 7 µM F-actin, 1 µM tropomyosin and 1 µM recombinant troponin complexes containing p.cTnC-G34S, or p.cTnI-D127Y, or wildtype subunits and incubated for 30 min at RT for stabilisation. The filaments were sedimented for 40 min at 67.000 x g and 4 °C. The proteins in supernatants and pellets of each sample were precipitated with 15 % (w/v) TCA, dissolved in 20 µl Laemmli sample buffer and analysed via SDS-PAGE. Mutant troponin variants were analysed pairwise with wildtype troponin on the same gel(s) to account for staining differences. Band intensities of Coomassie stained gels were determined using the GelDoc MP imager and the ImageLab software (Bio-Rad). The binding ratio was calculated from band intensities of the cTnI band (24 kDa; for p.cTnI-D127Y vs. WT) or the cTnC band (17 kDa; for p.cTnC-G34S vs. WT): B_rel=I_p/(I_P+I_S)*100"\%".

Brel is the ratio of the protein bound to the thin filament; IP and IS are the intensities of the corresponding gel band in the pellet and supernatant, respectively. For p.cTnI-D127Y a binding ratio of 41.51±1.63 % was determined, which was not significantly different from wildtype cTnI (45.21±1.99 %, p=0.15, *n*=34 each).
